# IBD-mediated oxidative cyclization of pyrimidinylhydrazones and concurrent Dimroth rearrangement: Synthesis of [1,2,4]triazolo[1,5-*c*]pyrimidine derivatives

**DOI:** 10.3762/bjoc.9.298

**Published:** 2013-11-25

**Authors:** Caifei Tang, Zhiming Li, Quanrui Wang

**Affiliations:** 1 Department of Chemistry, Fudan University, 200433 Shanghai, P. R. of China

**Keywords:** cyclization, hydrazones, hypervalent iodine, oxidation, rearrangement, [1,2,4]triazolo[1,5-*c*]pyrimidines

## Abstract

Oxidative cyclization of 6-chloro-4-pyrimidinylhydrazones **4** with iodobenzene diacetate (IBD) in dichloromethane gives rise to [1,2,4]triazolo[4,3-*c*]pyrimidine derivatives **5a**–**o**. These incipient products undergo feasible Dimroth rearrangement to furnish the isolated [1,2,4]triazolo[1,5-*c*]pyrimidines **6a**–**o** in moderate to high yields.

## Introduction

**Figure 1:**
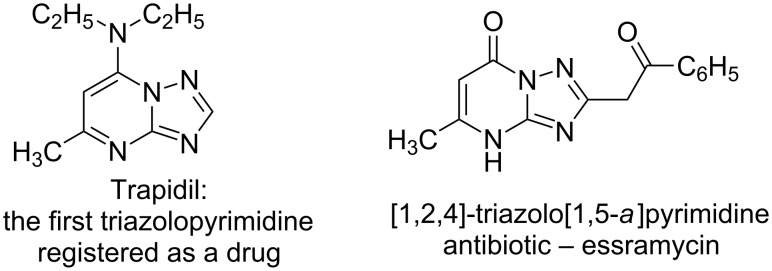
The structure of two representatives of [1,2,4]triazolopyrimidines.

The pyrimidine moiety is an important pharmacophore [1]. Especially, the fused bi- or tricyclic heterocyles containing a pyrimidine motif have received considerable interest in the design and discovery of new compounds for pharmaceutical and herbicidal applications [2–3]. For example, the pyrrolo[2,3-*d*]pyrimidine derivative, ruxolitinib (INCB018424), was discovered as a selective JAK1 and JAK2 inhibitor, which is currently under clinical investigation [4]. To date, a number of fused pyrimidine-type compounds have been successfully commercialized, such as the pyrazolo[3,4-*d*]pyrimidine allopurinol and the pyrazolo[4,3-*d*]pyrimidine sildenafil. In addition, trapidil is a [1,2,4]triazolo[1,5-*a*]pyrimidine compound, which is the first triazolopyrimidine registered as a drug possessing antiproliferative activity in glioma cells and vascular smooth muscle cells (VSMCs) and used as a coronary vasodilator in the therapy of syndromes that may be influenced by immunomodulators [5–6]. Interestingly, the first [1,2,4]triazolo[1,5-*a*]pyrimidine antibiotic essramycin has been isolated from natural sources (Figure 1) [7].

It has also been revealed that compounds carrying a [1,2,4]triazolo[1,5-*c*]pyrimidine nucleus behave as selective antagonists for human A_2A_ and A_3_ adenosine receptor sub-types which offer great promise in the treatment of Parkinson’s disease, or as Hsup90 modulators [8]. Clarkson and co-workers have prepared a range of benzothieno-fused 1,2,4-triazolo[4,3-*c*]pyrimidines and 1,2,4-triazolo[1,5-*c*]pyrimidines by the oxidative cyclisation of benzothieno[2,3-*d*]pyrimidine hydrazones. The investigation has also suggested the important ability as inhibitors of Shiga toxin trafficking for protecting HeLa cells [9].

The wide range of biological activities shown by various triazolopyrimidines encouraged synthetic organic and medicinal chemists to synthesize new members and to study their applications [10–11]. Several strategies for constructing triazolopyrimidine rings are available. These include the pre-formation of 1-amino-6-imino-substituted pyrimidines followed by reaction with carboxylic acids or derivatives as the one-carbon cyclizing agent [12] and the dehydrative cyclization of carboxylic acid *N*'-(pyrimidin-4-yl)-hydrazide and ring rearrangement [13]. Recently, Thiel and coworkers have also established an efficient palladium-catalyzed intermolecular coupling of aldehyde-derived hydrazones with chloro-substituted pyridines and related heterocycles, from which the triazolopyridines and other related fused heterocycles can be obtained after oxidative cyclization [14]. In accordance with the significance shown by triazolopyrimidines, the development of complementary and simple synthetic methods for the efficient preparation of novel triazolopyrimidines is needed.

Recently, we have reported the synthesis of 8-bromo-7-chloro-[1,2,4]triazolo[1,5-*c*]pyrimidines from the reaction of pyrimidinylhydrazones with bromine followed by Dimroth rearrangement [15]. In the present paper, we wish to report an efficient synthesis of new [1,2,4]triazolo[1,5-*c*]pyrimidine derivatives from hypervalent iodine (IBD)-mediated oxidative cyclisation of aldehyde pyrimidinylhydrazones and consecutive Dimroth rearrangement in relatively good yields under very mild conditions.

## Results and Discussion

The investigation commenced with the preparation of 1-(6-chloropyrimidin-4-yl)hydrazines **3**. The required starting 4,6-dihydroxypyrimidines **1** can be prepared following the reported procedure by condensation of dimethyl malonate either with formamide (for **1a**) or the respective formamidine salts (for **1b** and **1c)** [15].

**Scheme 1:**
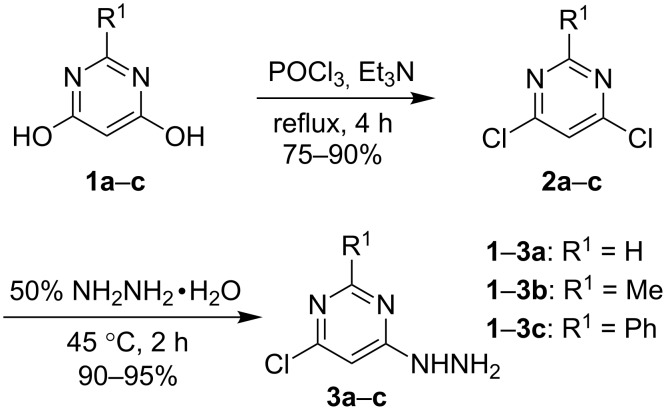
Synthesis of 1-(6-chloropyrimidin-4-yl)hydrazines **3**.

As outlined in Scheme 1, the conversion of 4,6-dihydroxypyrimidines **1** to the dichloro derivatives **2** was accomplished by refluxing in phosphorus oxychloride for 4 h in the presence of triethylamine in yields ranging from 75 to 90% [16–17]. After screening a set of conditions, 1-(6-chloropyrimidin-4-yl)hydrazines **3** were then obtained in 90–95% yield through the reaction of compounds **2** with two equivalents of 50% hydrazine in ethanol at 45 °C for 2 h (Scheme 1).

We next examined the synthesis of the corresponding hydrazones of various aldehydes. After screening a set of conditions, optimal conditions could be determined. Thus, 1-(6-chloropyrimidin-4-yl)hydrazine (**3a**) was condensed with 20% excess benzaldehyde to give 85% yield of the chloropyrimidinylhydrazone **4a** in absolute ethanol at room temperature in about 1 h. As shown in Table 1, the reaction proceeded readily for most of the employed aldehydes, affording the corresponding hydrazones **4**. In general, aromatic aldehydes provided higher yields than aliphatic aldehydes.

**Table 1:** Synthesis of the chloropyrimidinylhydrazones **4**^a^.

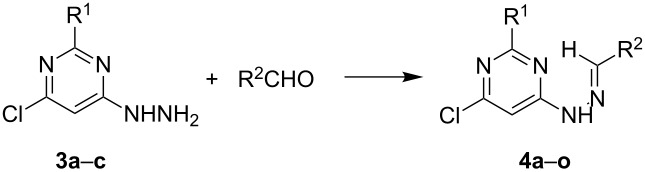				

Entry	R^1^	R^2^	Product	Yield [%]^b^

1	H	Ph	**4a**	85
2	H	2-ClC_6_H_4_	**4b**	84
3	H	2-furanyl	**4c**	75
4	H	4-(MeO)C_6_H_4_	**4d**	80
5	H	Et	**4e**	66
6	Me	Ph	**4f**	81
7	Me	2-ClC_6_H_4_	**4g**	85
8	Me	2-furanyl	**4h**	83
9	Me	4-(MeO)C_6_H_4_	**4i**	85
10	Me	Et	**4j**	84
11	Ph	Ph	**4k**	89
12	Ph	2-ClC_6_H_4_	**4l**	87
13	Ph	2-furanyl	**4m**	90
14	Ph	4-(MeO)C_6_H_4_	**4n**	85
15	Ph	Et	**4o**	96

^a^Reagents and conditions: chloropyriminylhydrazine **3** (3.0 mmol), EtOH (15 mL), aldehyde (3.6 mmol, 1.2 equiv), rt, 30–60 min. ^b^Isolated yield after column chromatography.

In recent years, organo hypervalent iodine reagents have drawn considerable interests as versatile and environmentally benign oxidants with many applications in organic synthesis because of their low toxicity, stability, high reactivity, and availability. Efficient formation of new carbon–heteroatom bonds as well as carbon–carbon bonds can be achieved typically under mild reaction conditions with these metal-free reagents [18–19]. For example, Salgado has described the synthesis of novel [1,2,4]triazolo[1,5-*a*]pyrimidine derivatives by iodobenzenediacetate (IBD)-mediated oxidative cyclization of suitable *N*-benzylidene-*N*′-pyrimidin-2-yl hydrazine precursors, followed by a Dimroth rearrangement [20]. We envisioned that our aldehyde chloropyrimidinylhydrazones **4** would undergo feasible oxidative cyclisation to afford novel triazolopyrimidines.

We initiated our investigation by examining the oxidative cyclization of **4a** with iodobenzenediacetate (IBD) as an oxidant (Table 2). Thus, **4a** was treated with one equivalent of freshly prepared IBD in dichloromethane at room temperature. After completion of the reaction, the solvent was removed and the residue was dissolved in EtOH with a catalytic amount of conc. HCl. Usual workup afforded the [1,2,4]triazolo[1,5-*c*]pyrimidine **6a** in 57% yield (Table 2, entry 1).

**Table 2:** Selected results of screening the optimal conditions.

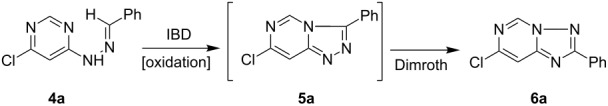						

Entry	IBD (equiv)	Additive	*T* (°C)	Time (h)	Solvent	Yield [%]^a^

1	1.0	–	25	4	CH_2_Cl_2_	57
2	1.1	–	25	4	CH_2_Cl_2_	66
3	1.2	–	25	4	CH_2_Cl_2_	75
4	1.3	–	25	4	CH_2_Cl_2_	75
5	1.3	–	25	6	CH_2_Cl_2_	81
6	1.3	–	25	24	CH_2_Cl_2_	79
7	1.3	–	40	10	CH_2_Cl_2_	72
8	1.3	–	25	6	THF	70
9	1.3	–	25	6	EtOAc	70
10	1.3	–	25	6	H_2_O	12^b^
11	1.3	–	25	6	EtOH	25
12	1.3	–	25	6	DMF	^c^
13	1.3	Bu_4_NI^d^	25	6	CH_2_Cl_2_	75
14	1.3	I_2_^d^	25	6	CH_2_Cl_2_	75

^a^Isolated yields of **6a**. ^b^Performed under microwave irradiation. ^c^Complex mixture of products. ^d^An amount of 10 mol % was used.

With this promising result in hand, the reaction efficiency was improved by optimizing the conditions, such as the amount of IBD, solvent, reaction time and purification method. Gratifyingly, **6a** was formed in 81% yield when 1.3 equiv of IBD were used in dichloromethane for 6 h (Table 2, entry 5). Further extension of the reaction time to 24 h did not provide superior results (Table 2, entry 6). Meanwhile, reactions in THF or ethyl acetate resulted in marginally lower yields (Table 2, entries 8 and 9). Switching to water with assistance by microwave irradiation or performing the reaction in ethanol provided even unacceptable yields (Table 2, entries 10 and 11). Further, when replacing the solvent by DMF, a complex mixture of products was produced, and no pure desired product could be isolated (Table 2, entry 12). Employment of 10 mol % Bu_4_NI or I_2_ as an additive did not improve the result (Table 2, entries 13 and 14). It has also been observed that temperature elevation did not benefit the transformation (Table 2, entry 7). Thus, the use of IBD (1.3 equiv) at room temperature in dichloromethane for 6 h was found to be most efficient and therefore used as the standard conditions.

To probe the generality of the IBD-oxidative cyclization protocol, all of the other pyrimidinylhydrazones **4** with different R^1^ and R^2^ groups were examined under optimal conditions. As shown in Table 3, the protocol appeared to be quite general. In all cases studied, the respective [1,2,4]triazolo[1,5-*c*]pyrimidines **6** could be obtained. Various aryl substituents on the triazole ring (R^2^), including phenyl, electron-rich and electron-poor aryls, were compatible with this reaction, and high yields of the triazolopyrimidines **6** could generally be obtained. Substrates carrying a heteroaromatic group such as 2-furanyl also proceeded smoothly to give a good yield of the product. Aromatic aldehyde hydrazones bearing an electron-donating functional group on the *para* position of the aromatic ring seems to afford slightly lower yields (Table 3, entries 4, 9, 14).

**Table 3:** Synthesis of the triazolopyrimidines **6**^a^.

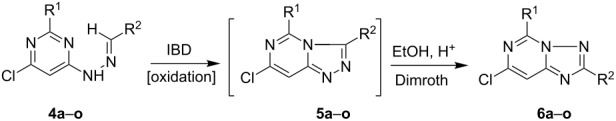				

Entry	R^1^	R^2^	Product	Yield [%]^b^

1	H	Ph	**6a**	81
2	H	2-ClC_6_H_4_	**6b**	80
3	H	2-furanyl	**6c**	81
4	H	4-(MeO)C_6_H_4_	**6d**	69
5	H	Et	**6e**	64
6	Me	Ph	**6f**	81
7	Me	2-ClC_6_H_4_	**6g**	86
8	Me	2-furanyl	**6h**	86
9	Me	4-(MeO)C_6_H_4_	**6i**	71
10	Me	Et	**6j**	34
11	Ph	Ph	**6k**	84
12	Ph	2-ClC_6_H_4_	**6l**	87
13	Ph	2-furanyl	**6m**	87
14	Ph	4-(MeO)C_6_H_4_	**6n**	71
15	Ph	Et	**6o**	36

^a^Reagents and conditions: hydrazone **4** (1.0 mmol), IBD (1.3 mmol), DCM (6 mL) at rt, then refluxed in abs. EtOH for 1–3 h. ^b^Isolated yield after column chromatography.

On the other hand, the reaction is less sensitive to substitution of **4** at the C-2 position. Although both aromatic and aliphatic aldehyde-derived hydrazones **4** participated well in the reaction, the latter furnished only low to moderate yields (Table 3, entries 5, 10 and 15).

**Scheme 2:**
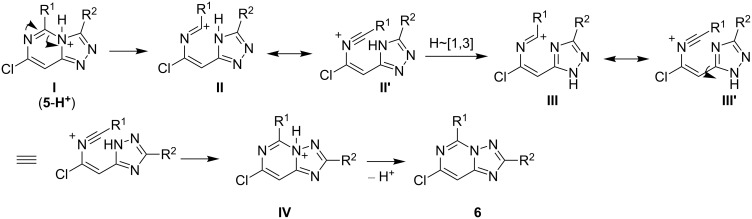
Plausible mechanism for the transformation of [1,2,4]triazolo[4,3-*c*]pyrimidines **5** to the [1,5-*c*] series **6**.

Although the employed procedure was believed to afford initially [1,2,4]triazolo[4,3-*c*]pyrimidines **5**, a formal Dimroth rearrangement occurred readily to give the isolated products that belong to the [1,5-*c*] series **6**. This kind of rearrangement has been described previously [10–11,21]. The accepted mechanism of the Dimroth rearrangement might involve protonation of **5** to give **I** (**5-H^+^**), ring opening (**II**), tautomerization by H-shift (**III**) of the 1,2,4-triazole ring, ring closure (**IV**) and deprotonation to isomeric 1,2,4-triazolo[1,5-*c*] pyrimidine **6** (Scheme 2).

Indeed, small quantities of the triazole **6** can be observed by ^1^H NMR and thin-layer chromatography in our original attempts to isolate pure **5**. Although very slow, the rearrangement of the triazole **5** to **6** was found to occur spontaneously. The process can be catalyzed under catalytic amount of HCl. In one case, pure 1,2,4-triazolo[4,3-*c*]pyrimidine **5f** was isolated and characterized (see Supporting Information File 1 for full experimental data), which was dissolved in ethanol and allowed to be stirred at room temperature. After one day, formation of **6f** was observed. The isomerization was completed after 10 days as evidenced by the disappearance of the methyl signal at 2.39 ppm in **5f** and fully shifting to downfield absorption at 3.04 ppm in **6f**.

Since compounds **5** are generally unstable as compared with their [1,5-*c*]-counterparts, isolation of the initial [1,2,4]triazolo[4,3-*c*]pyrimidine intermediates proved to be of no preparative value. Instead, they were allowed directly to isomerize under HCl catalysis to provide the thermodynamically more stable title compounds through tandem ring opening and ring closing Dimroth reactions.

The structure of bicycles **6a**–**o** was principally assigned from their spectral evidence and mass spectrometry analysis (see Supporting Information File 2 for full data). To confirm the structure of the regio-isomer of the cyclization rearrangement, colorless single crystals of compound **6h** were obtained from CH_2_Cl_2_/*n*-hexane and its molecular structure was determined by X-ray crystallography, providing unequivocal evidence for the assignments [22].

## Conclusion

In conclusion, we report herein a general and convenient method for the synthesis of novel [1,2,4]triazolo[1,5-*c*]pyrimidine derivatives with moderate to excellent yields. The protocol features initial oxidative cyclization of readily available chloropyrimidinylhydrazones followed by feasible Dimroth rearrangement. The procedure offers several advantages including good yields, operational simplicity, environmental friendliness and shorter reaction time along with broad substrate scope, which make it a useful and attractive process for the synthesis of structurally diverse triazolopyrimidines. Moreover, the 7-chloro-substitution would allow further manipulations through nucleophilic substitution [23] and metal-catalyzed cross-coupling reactions, which are under investigation in our group.

## Supporting Information

File 1 Experimental procedures and analytical data for new compounds.

File 2 NMR spectral data for unknown compounds.
