# Supplementary material for: IBD-mediated oxidative cyclization of pyrimidinylhydrazones and concurrent Dimroth rearrangement: Synthesis of [1,2,4]triazolo[1,5-c]pyrimidine derivatives
Source: Beilstein J Org Chem. 2013 Nov 25;9:2629–34. doi: 10.3762/bjoc.9.298 (PMC3869367; doi:10.3762/bjoc.9.298)
Supplement: File 1 — Experimental procedures and analytical data for new compounds. [file Beilstein_J_Org_Chem-09-2629-s001.pdf]

Supporting Information

for

IBD-mediated oxidative cyclization of pyrimidinylhydrazones and concurrent Dimroth rearrangement: Synthesis of [1,2,4]triazolo[1,5-*c*]pyrimidine derivatives

Caifei Tang, Zhiming Li and Quanrui Wang

Address: Department of Chemistry, Fudan University, 220 Handan Road, 200433 Shanghai, P. R. China

Email: Quanrui Wang* - qrwang@fudan.edu.cn

*Corresponding author

Experimental procedures and analytical data for new compounds

1. General Information……………………………………………………………S2
2. Preparation of chloropyrimidinylhydrazones (**4a**–**o**); general procedure…………………………………………………………………… S3
3. Preparation of 7-chloro-5-methyl-3-phenyl[1,2,4]triazolo[4,3-*c*]-pyrimidine **5f** ……………………………………………………………………S9
4. 2,5-Disubstituted 7-chloro-[1,2,4]triazolo[1,5-*c*]pyrimidines (**6a**–**o**); general procedure…………………………………………………………… S10
5. References…………………………………………………………………… S17

1. General information: Nuclear magnetic resonance spectra were taken on a JEOLECA (^1H, 400 MHz; ^{13}C, 100 MHz) spectrometer using tetramethylsilane (^1H) as an internal standard or with respect to the residual protons of the deuterated solvent: $CDCl_3$ (7.26, 77.16 ppm), DMSO-d_6 (2.50, 39.52 ppm). ^1H NMR data are reported as follows: chemical shift (δ, ppm), multiplicity (s = singlet, d = doublet, t = triplet, q = quartet, m = multiplet, bs = broad singlet), coupling constants (Hz), integration, and identification. High-resolution mass spectra were obtained with a Bruker Micro TOF 11 spectrometer at the positive electrospray ionization (ESI) mode. IR spectra were recorded in KBr disks using a Nicolet 360 spectrometer. TLC analyses were performed by means of Huanghai F254 (0.25 mm) Plates. Visualization was accomplished with UV light (254 nm). Column chromatography was carried out using Huanghai silica gel (spherical, 200–300 mm). Microwave reactions were carried out in a microwave oven with a 2500 W power (Micro-Synth, Milestone).

All reagents were purchased from commercial suppliers and were used without further purification. IBD was prepared according to the literature procedure [1]. The substrates of 4,6-dihydroxypyrimidines **1**, 4,6-dichloropyrimidines **2** and the chloropyrimidinylhydrazines **3** were prepared according to known procedures [2,3].

2. Preparation of chloropyrimidinylhydrazones (4a–o); general procedure

To the suspension of appropriate 1-(6-chloropyrimidin-4-yl)hydrazine (**3**) (3.0 mmol) in EtOH (15 mL) was added dropwise with vigorous stirring an aldehyde (3.6 mmol, 1.2 equiv) at rt over 0.5–1 h. The progression of the reaction was monitored by TLC. After completion of the reaction, the solvent was removed under reduced pressure to afford the crude product. Crystallization from an appropriate solvent or purification by column chromatography on silica gel furnished the pure hydrazones **4**.

Benzaldehyde (6-chloro-4-pyrimidinyl)hydrazone (4a) [2]

Yield: 0.59 g (85%); white powder.

^{1}H NMR (400 MHz, $CDCl_3$): δ = 8.80 (s, 1 H, NH), 8.46 (s, 1 H, Ar-H), 7.85 (s, 1 H, CH), 7.72-7.69 (m, 2 H, Ar-H), 7.46-7.42 (m, 3 H, Ar-H), 7.30 (d, J = 0.4 Hz, 1 H, Ar-H).

2-Chlorobenzaldehyde (6-chloro-4-pyrimidinyl)hydrazone (4b) [2]

Yield: 0.67 g (84%); light yellow powder.

^{1}H NMR (400 MHz, DMSO-d_6): δ = 12.00 (s, 1H, NH), 8.55 (s, 1 H, Ar-H), 8.46 (s, 1 H, CH), 8.16-8.13 (m, 1 H, Ar-H), 7.52-7.49 (m, 1 H, Ar-H), 7.45-7.39 (m, 2 H, Ar-H), 7.28 (s, 1 H, Ar-H).

Furfural (6-chloro-4-pyrimidinyl)hydrazone (4c)

Yield: 0.50 g (75%); yellow powder; mp 220-221 °C.

IR (KBr): 3209, 3070, 1627, 1591, 1443, 1119, 1078, 985, 801, 713 cm^{-1}.

^1H NMR (400 MHz, DMSO-d_6): δ = 11.79 (s, 1 H, NH), 8.43 (s, 1 H, Ar-H), 8.04 (s, 1 H, CH), 7.83 (s, 1 H, Ar-H), 7.03 (s, 1 H, Ar-H), 6.94 (d, J = 2.8 Hz, 1 H, Ar-H), 6.63-6.62 (m, 1 H, Ar-H).

^{13}C NMR (100 MHz, DMSO-d_6): δ = 162.1, 159.4, 158.5, 149.3, 145.1, 134.6, 113.2, 112.3, 101.4. HRMS (ESI): *m/z* $[M + Na]^+$ *calcd* for $C_9H_7N_4ClO$: 245.0206; found: 245.0202.

4-Methoxybenzaldehyde (6-chloro-4-pyrimidinyl)hydrazone (4d) [2]

Yield: 0.63 g (80%); white powder.

^1H NMR (400 MHz, $CDCl_3$): δ = 8.97 (s, 1 H, NH), 8.43 (s, 1 H, Ar-H), 7.80 (s, 1 H, CH), 7.64 (d, J = 7.2 Hz, 2 H, Ar-H), 7.27 (s, 1 H, Ar-H), 6.95 (d, J = 7.2 Hz, 2 H, Ar-H), 3.86 (s, 3 H, CH_3).

Propanaldehyde (6-chloro-4-pyrimidinyl)hydrazone (4e) [2]

Yield: 0.36 g (66%); white powder.

^1H NMR (400 MHz, DMSO-d_6): δ = 11.39 (s, 1 H, NH), 8.36 (s, 1 H, Ar-H), 7.51 (t, J = 4.0 Hz, 1 H, CH), 6.91 (s, 1 H, Ar-H), 2.30-2.25 (m, 2 H, CH_2), 1.05 (t, J = 6.0 Hz, 3 H, CH_3).

Benzaldehyde (6-chloro-2-methyl-4-pyrimidinyl)hydrazone (4f)

Yield: 0.60 g (81%); white powder; mp 165-167 °C.

IR (KBr): 3210, 3064, 1604, 1584, 1407, 1370, 1122, 977, 862, 835 cm^{-1}.

^1H NMR (400 MHz, $CDCl_3$): δ = 8.78 (s, 1 H, NH), 7.81 (s, 1 H, CH), 7.70-7.68 (m, 2 H, Ar-H), 7.45-7.39 (m, 3 H, Ar-H), 7.14 (s, 1 H, Ar-H), 2.54 (s, 3 H, CH_3).

^{13}C NMR (100 MHz, $CDCl_3$): δ = 168.1, 162.2, 160.7, 143.9, 133.7, 130.3, 128.9, 127.2, 100.3, 25.6.

HRMS (ESI): *m/z* $[M + H]^+$ *calcd* for $C_{12}H_{11}N_4Cl$: 247.0750; found: 247.0765.

2-Chlorobenzaldehyde (6-chloro-2-methyl-4-pyrimidinyl)hydrazone (4g)

Yield: 0.72 g (85%); pale yellow powder. mp = 191-193 °C.

IR (KBr, v/cm-1): 3199, 3060, 1602, 1550, 1407, 1145, 980, 831, 754 cm^{-1}.

^{1}H NMR (400 MHz, $CDCl_3$): δ = 9.19 (s, 1 H, NH), 8.24 (s, 1 H, CH), 8.06-8.03 (m, 1 H, Ar-H), 7.39-7.36 (m, 1 H, Ar-H), 7.33-7.30 (m, 2 H, Ar-H), 7.13 (s, 1 H, Ar-H), 2.55 (s, 3 H, CH_3).

^{13}C NMR (100 MHz, $CDCl_3$): δ = 168.2, 162.2, 160.9, 140.3, 133.9, 131.2, 131.0, 130.1, 127.2, 127.1, 100.5, 25.6.

HRMS (ESI): *m/z* $[M + H]^+$ *calcd* for $C_{12}H_{10}N_4Cl_2$: 281.0361; found: 281.0374.

Furfural (6-chloro-2-methyl-4-pyrimidinyl)hydrazone (4h)

Yield: 0.59 g (83%); yellow powder; mp 167-169 °C.

IR (KBr): 3204, 3065, 1622, 1581, 1407, 1129, 1021, 975, 826, 795 cm^{-1}.

^{1}H NMR (400 MHz, $CDCl_3$): δ = 8.68 (s, 1 H, NH), 7.68 (s, 1 H, CH), 7.54-7.53 (m, 1 H, Ar-H), 7.10 (d, J = 0.4 Hz, 1 H, Ar-H), 6.72 (d, J = 3.2 Hz, 1 H, Ar-H), 6.50 (q, J = 1.6 Hz, 1 H, Ar-H), 2.52 (s, 3 H, CH_3).

^{13}C NMR (100 MHz, $CDCl_3$): δ = 168.0, 162.0, 160.8, 149.1, 144.7, 133.5, 112.8, 112.1, 100.3, 25.6.

HRMS (ESI): *m/z* $[M + H]^+$ *calcd* for $C_{10}H_9N_4ClO$: 237.0543; found: 237.0557.

4-Methoxybenzaldehyde (6-chloro-2-methyl-4-pyrimidinyl)hydrazone (4i)

Yield: 0.71 g (85%); white powder; mp 175-176 °C.

IR (KBr): 3183, 3055, 1591, 1555, 1509, 1407, 1247, 1165, 1032, 826 cm^{-1}.

^1H NMR (400 MHz, $CDCl_3$): δ = 8.70 (s, 1 H, NH), 7.74 (s, 1 H, CH), 7.62 (d, *J* = 8.8 Hz, 2 H, Ar-H), 7.09 (s, 1 H, Ar- H), 6.93 (d, *J* = 8.8 Hz, 2 H, Ar-H), 3.85 (s, 3 H, CH_3), 2.52 (s, 3 H, CH_3).

^{13}C NMR (100 MHz, $CDCl_3$): δ = 168.0, 162.2, 161.4, 160.6, 143.7, 128.7, 126.4, 114.4, 100.1, 55.5, 25.7.

HRMS (ESI): *m/z* $[M + H]^+$ *calcd* for $C_{13}H_{13}N_4ClO$: 277.0856; found: 277.0859.

Propanaldehyde (6-chloro-2-methyl-4-pyrimidinyl)hydrazone (4j)

Yield: 0.50 g (84%); white powder; mp 135-137 °C.

IR (KBr): 3203, 3065, 2973, 1591, 1407, 1371, 1227, 975, 872, 831 cm^{-1}.

^1H NMR (400 MHz, $CDCl_3$): δ = 8.21 (s, 1 H, NH), 7.20 (t, *J* = 5.0 Hz, 1 H, CH), 6.96 (s, 1 H, Ar-H), 2.50 (s, 3 H, CH_3), 2.39-2.32 (m, 2 H, CH_2), 1.15 (t, *J* = 7.4 Hz, 3 H, CH_3).

^{13}C NMR (100 MHz, $CDCl_3$): δ = 168.0, 162.4, 160.4, 148.7, 99.8, 25.8, 25.6, 10.8.

HRMS (ESI): *m/z* $[M + H]^+$ *calcd* for $C_8H_{11}N_4Cl$: 199.0750; found: 199.0755.

Benzaldehyde (6-chloro-2-phenyl-4-pyrimidinyl)hydrazone (4k)

Yield: 0.82 g (89%); white powder; mp 200-202 °C.

IR (KBr): 3190, 3045, 1604, 1448, 1391, 1160, 1068, 981, 830, 749 cm^{-1}.

^{1}H NMR (400 MHz, $CDCl_3$): δ = 9.03 (s, 1 H, NH), 8.37 (t, J = 3.6 Hz, 2 H, Ar-H), 7.67 (s, 1 H, CH), 7.63 (d, J = 5.2 Hz, 2 H, Ar-H), 7.48-7.40 (m, 6 H, Ar-H), 7.21 (s, 1 H, Ar-H).

^{13}C NMR (100 MHz, $CDCl_3$): δ = 164.7, 162.7, 161.4, 143.8, 136.5, 133.7, 131.4, 130.2, 128.84, 128.77, 128.5, 127.1, 101.2.

HRMS (ESI): *m/z calcd* for $C_{17}H_{13}N_4Cl$ $[M + H]^+$ 309.0907; found: 309.0930.

2-Chlorobenzaldehyde (6-chloro-2-phenyl-4-pyrimidinyl)hydrazone (4l)

Yield: 0.90 (87%); light yellow powder; mp 173-175 °C.

IR (KBr): 3189, 3060, 1622, 1386, 1231, 1088, 924, 821, 744 cm^{-1}.

^{1}H NMR (400 MHz, $CDCl_3$): δ = 8.94 (s, 1 H, NH), 8.39-8.36 (m, 2 H, Ar-H), 8.24 (s, 1 H, CH), 8.08-8.05 (m, 1 H, Ar-H), 7.49-7.44 (m, 3 H, Ar-H), 7.40-7.37 (m, 1 H, Ar-H), 7.36-7.30 (m, 2 H, Ar-H), 7.19 (s, 1 H, Ar-H).

^{13}C NMR (100 MHz, $CDCl_3$): δ = 164.6, 162.4, 161.4, 140.0, 136.3, 133.9, 131.4, 131.2, 131.0, 130.0, 128.6, 128.5, 127.2, 127.1, 101.2.

HRMS (ESI): *m/z* [M + H]+ *calcd* for $C_{17}H_{12}N_4Cl_2$: 343.0517; found: 343.0521.

Furfural (6-chloro-2-phenyl-4-pyrimidinyl)hydrazone (4m)

Yield: 0.70 g (90%); yellow powder; mp 167-169 °C.

IR (KBr): 3187, 3057, 1632, 1587, 1382, 1230, 1163, 1087, 1016, 828 cm^{-1}.

^{1}H NMR (400 MHz, $CDCl_3$): δ = 9.27 (bs), 9.20 (bs) (1 H, NH), 8.34 (t, J = 3.6 Hz, 2 H, Ar-H), 7.51-7.37 (m, 5 H, Ar-H, CH), 7.19 (s, 1 H, Ar-H), 6.58 (s, 1 H, Ar-H), 6.47 (s, 1 H, Ar-H).

^{13}C NMR (100 MHz, $CDCl_3$): δ = 164.6, 162.6, 161.5, 149.1, 144.5, 136.5, 133.6, 131.4, 128.8, 128.5, 113.0, 112.0, 101.2.

HRMS (ESI): *m/z* $[M + H]^+$ *calcd* for $C_{15}H_{11}N_4ClO$: 299.0700; found: 299.0722.

4-Methoxybenzaldehyde (6-chloro-2-phenyl-4-pyrimidinyl)hydrazone (4n)

Yield: 0.86 g (85%); white powder; mp 206-208 °C.

IR (KBr): 3178, 3070, 1581, 1550, 1504, 1386, 1274, 1165, 831 cm^{-1}.

^{1}H NMR (400 MHz, $CDCl_3$): δ = 8.95 (s, 1 H, NH), 8.36 (t, J = 3.6 Hz, 2 H, Ar-H), 7.61 (s, 1 H, CH), 7.57 (d, J = 8.4 Hz, 2 H, Ar-H), 7.46 (d, J = 4.0 Hz, 3 H, Ar-H), 7.17 (s, 1 H, Ar-H), 6.93 (d, J = 8.4 Hz, 2 H, Ar-H), 3.85 (s, 3 H, CH_3).

^{13}C NMR (100 MHz, $CDCl_3$): δ = 164.6, 162.7, 161.2, 143.8, 136.6, 131.4, 128.7, 128.6, 128.5, 126.5, 114.3, 100.9, 55.5.

HRMS (ESI): *m/z calcd* for $C_{18}H_{15}N_4ClO$ $[M + H]^+$ 339.1013; found: 339.1042.

Propanaldehyde (6-chloro-2-phenyl-4-pyrimidinyl)hydrazone (4o)

Yield: 0.75 g (96%); white powder; mp 128-130 °C.

IR (KBr): 3194, 3060, 1581, 1545, 1391, 1232, 1078, 831, 698 cm^{-1}.

^{1}H NMR (400 MHz, $CDCl_3$): δ = 8.89 (s, 1 H, NH), 8.38-8.31 (m, 2 H, Ar-H), 7.45 (d, J = 7.2 Hz, 3 H, Ar-H), 7.04 (s, 1 H, Ar-H), 7.01-7.00 (m, 1 H, CH), 2.27-2.22 (m, 2 H, CH_2), 1.06 (t, J = 7.4 Hz, 3 H, CH_3).

^{13}C NMR (100 MHz, $CDCl_3$): δ = 164.6, 162.8, 161.1, 149.0, 136.6, 131.2, 128.7, 128.4, 100.6, 25.7, 10.6.

HRMS (ESI): *m/z* $[M + H]^+$ *calcd* for $C_{13}H_{13}N_4Cl$: 261.0907; found: 261.0927.

3. Preparation of 7-chloro-5-methyl-3-phenyl-[1,2,4]triazolo[4,3-*c*] pyrimidine (5f): Iodobenzenediacetate (IBD) (0.42 g, 1.3 mmol, 1.3 equiv) was added to a suspension of hydrazone **4f** (1.00 mmol, 1.0 equiv) in dichloromethane, and the mixture was stirred at rt for 6 h. The reaction was monitored by TLC. After completion of the reaction, the mixture was concentrated under reduce pressure, and the residue was purified by column chromatography on silca gel eluting with PE:EA to give pure **5f**.

Yield: 0.22 g (90 %); white powder; mp 130-132 °C.

IR (KBr): 3070, 3035, 1614, 1529, 1422, 1266, 1172, 1092, 967 cm^{-1}.

^{1}H NMR (400 MHz, $CDCl_3$): δ = 7.62-7.58 (m, 2 H, Ar-H), 7.57-7.51 (m, 4 H, Ar-H), 2.39 (s, 3H, CH_3).

^{13}C NMR (100 MHz, $CDCl_3$): δ = 151.1, 148.5, 146.5, 144.2, 131.1, 130.9, 128.7, 127.6, 107.5, 23.4.

HRMS (ESI): *m/z* $[M + H]^+$ *calcd* for $C_{12}H_9N_4Cl$: 245.0594; found: 245.0592.

4. 2,5-Disubstituted 7-chloro-[1,2,4]triazolo[1,5-*c*]pyrimidines (6a–o); general procedure

Iodobenzenediacetate (IBD) (0.42 g, 1.3 mmol) was added to a suspension of appropriate hydrazone **4** (1.0 mmol) in dichloromethane, and the mixture was stirred at rt for 6–8 h. The progression of the reaction was monitored by TLC. After completion of the reaction, the solvent was removed under reduced pressure, and the residue was dissolved in EtOH (15 mL) containing a catalytic amount conc. HCl. Then the mixture was concentrated under reduced pressure, and the residue was purified by column chromatography on silca gel eluting with PE:EA to give the corresponding products **6**.

7-Chloro-2-phenyl-[1,2,4]triazolo[1,5-*c*]pyrimidine (6a)

Yield: 0.19 g (81%); white powder; mp 228-229 °C.

IR (KBr): 3061, 1619, 1445, 1395, 1365, 1266, 1066, 714 cm^{-1}.

^{1}H NMR (400 MHz, $CDCl_3$): δ = 9.28 (s, 1 H, C(5)-H), 8.30 (s, 2 H, Ar-H), 7.75 (s, 1 H, Ar-H), 7.54 (s, 3 H, Ar-H).

^{13}C NMR (100 MHz, $CDCl_3$): δ = 167.6, 153.8, 148.0, 140.0, 131.5, 129.4, 129.1, 128.0, 110.5.

HRMS (ESI): *m/z* $[M + H]^+$ *calcd* for $C_{11}H_7N_4Cl$: 231.0437; found: 231.0433.

7-Chloro-2-(2-chlorophenyl)-[1,2,4]triazolo[1,5-*c*]pyrimidine (6b)

Yield: 0.21 g (80%); light yellow needle crystals; mp 168-169 °C.

IR (KBr): 3060, 3036, 1621, 1496, 1443, 1347, 1265, 1063, 1044, 938 cm^{-1}.

^{1}H NMR (400 MHz, $CDCl_3$): δ = 9.33 (d, J = 1.2 Hz, 1 H, C(5)-H), 8.05-8.03 (m, 1 H, Ar-H), 7.75 (d, J = 1.2 Hz, 1 H, Ar-H), 7.58-7.56 (m, 1 H, Ar-H), 7.48-7.40 (m, 2 H, Ar-H).

^{13}C NMR (100 MHz, $CDCl_3$): δ = 164.5, 152.7, 146.8, 142.1, 132.22, 132.17, 132.0, 130.9, 128.4, 127.5, 110.4.

HRMS (ESI): *m/z* $[M + H]^+$ *calcd* for $C_{11}H_6N_4Cl_2$: 265.0048; found: 265.0071.

7-Chloro-2-(2-furanyl)-[1,2,4]triazolo[1,5-*c*]pyrimidine (6c)

Yield: 0.18 g (81%); yellow powder; mp 216-218 °C.

IR (KBr): 3070, 3040, 1617, 1504, 1432, 1355, 1186, 1078, 1016, 939 cm^{-1}.

^{1}H NMR (400 MHz, $CDCl_3$): δ = 9.23 (s, 1 H, C(5)-H), 7.66 (s, 2 H, Ar-H), 7.29 (d, J = 2.4 Hz, 1 H, Ar-H), 6.62-6.61 (m, 1 H, Ar-H).

^{13}C NMR (100 MHz, $CDCl_3$): δ = 160.0, 153.7, 148.5, 145.6, 145.0, 140.0, 114.1, 112.3, 110.4.

HRMS (ESI): *m/z* $[M + H]^+$ *calcd* for $C_9H_5N_4ClO$: 221.0230; found: 221.0239.

7-Chloro-2-(4-methoxyphenyl)-[1,2,4]triazolo[1,5-*c*]pyrimidine (6d)

Yield: 0.18 g (69%); white powder; mp 230-232 °C.

IR (KBr): 3039, 3011, 2966, 1622, 1578, 1457, 1392, 1251, 1057, 940 cm^{-1}.

^{1}H NMR (400 MHz, $CDCl_3$): δ = 9.21 (d, J = 0.8 Hz, 1 H, C(5)-H), 8.21 (d, J = 7.2 Hz, 2 H, Ar-H), 7.64 (d, J = 1.2 Hz, 1 H, Ar-H), 7.02 (d, J = 7.2 Hz, 2 H, Ar-H), 3.89 (s, 3 H, CH_3).

^{13}C NMR (100 MHz, $CDCl_3$): δ = 167.5, 162.3, 153.8, 147.9, 139.8, 129.6, 121.9, 114.4, 110.1, 55.6.

HRMS (ESI): *m/z* $[M + H]^+$ *calcd* for $C_{12}H_9N_4ClO$: 261.0543; found: 261.0550.

7-Chloro-2-ethyl-[1,2,4]triazolo[1,5-*c*]pyrimidine (6e)

Yield: 0.12 g (64%); white powder; mp 160-162 °C.

IR (KBr): 3045, 2978, 1617, 1519, 1432, 1278, 1186, 1083, 903 cm^{-1}.

^1H NMR (400 MHz, DMSO-d_6): δ = 9.69 (d, J = 1.2 Hz, 1 H, C(5)-H), 8.04 (d, J = 1.6 Hz, 1 H, Ar-H), 2.88 (q, J = 8.0 Hz, 2 H, CH_2), 1.32 (t, J = 8.0 Hz, 3 H, CH_3).

^{13}C NMR (100 MHz, DMSO-d_6): δ = 171.1, 152.9, 146.3, 141.6, 109.7, 21.7, 11.9.

HRMS (ESI): *m/z* $[M + H]^+$ *calcd* for $C_7H_7N_4Cl$: 183.0437; found: 183.0453.

7-Chloro-5-methyl-2-phenyl-[1,2,4]triazolo[1,5-*c*]pyrimidine (6f)

Yield: 0.20 g (81%); white powder; mp 190-192 °C.

IR (KBr): 3061, 3015, 1622, 1534, 1427, 1356, 1221, 1113, 970, 905 cm^{-1}.

^1H NMR (400 MHz, $CDCl_3$): δ = 8.32-8.29 (m, 2 H, Ar-H), 7.57 (s, 1 H, Ar-H), 7.53-7.50 (m, 3 H, Ar-H), 3.05 (s, 3 H, CH_3).

^{13}C NMR (100 MHz, $CDCl_3$): δ = 166.4, 153.9, 151.2, 147.2, 131.2, 129.7, 128.9, 127.9, 108.1, 19.9.

HRMS (ESI): *m/z* $[M + H]^+$ *calcd* for $C_{12}H_9N_4Cl$: 245.0594; found: 245.0614.

7-Chloro-2-(2-chlorophenyl)-5-methyl-[1,2,4]triazolo[1,5-*c*]pyrimidine (6g)

Yield: 0.24 g (86%); pale yellow powder; mp 171-172 ° C.

IR (KBr): 3050, 2925, 1617, 1525, 1414, 1374, 1111, 1049, 900, 856 cm^{-1}.

^{1}H NMR (400 MHz, $CDCl_3$): δ = 7.65 (s, 1 H, C(8)-H), 7.62-7.57 (m, 3 H, Ar-H), 7.50-7.46 (m, 1 H, Ar-H), 2.43 (s, 3 H, CH_3).

^{13}C NMR (100 MHz, $CDCl_3$): δ = 165.1, 153.2, 151.4, 147.4, 133.6, 132.3, 131.5, 131.1, 129.0, 127.0, 108.4, 19.9.

HRMS (ESI): *m/z* $[M + H]^+$ *calcd* for $C_{12}H_8N_4Cl_2$: 279.0204; found: 279.0239.

7-Chloro-2-(2-furanyl)-5-methyl-[1,2,4]triazolo[1,5-*c*]pyrimidine (6h)

Yield: 0.20 g (86%); pale yellow powder; mp 184-185 °C.

IR (KBr): 3095, 3051, 1747, 1614, 1533, 1420, 1352, 1299, 1178, 1021, 981 cm^{-1}.

^{1}H NMR (400 MHz, $CDCl_3$): δ = 7.62 (s, 1 H, C(8)-H), 7.49 (s, 1 H, Ar-H), 7.23 (d, *J* = 3.2 Hz, 1 H, Ar-H), 6.57 (d, *J* = 1.2 Hz, 1 H, Ar-H), 3.00 (s, 3 H, CH_3).

^{13}C NMR (100 MHz, $CDCl_3$): δ = 158.9, 153.8, 151.2, 147.8, 145.3, 145.2, 113.7, 112.2, 108.0, 20.0.

HRMS (ESI): *m/z* $[M + H]^+$ *calcd* for $C_{10}H_7N_4ClO$: 235.0387; found: 235.0408.

7-Chloro-2-(4-methoxyphenyl)-5-methyl-[1,2,4]triazolo[1,5-*c*]pyrimidine (6i)

Yield: 0.19 g (71%); white powder; mp 186-188 °C.

IR (KBr): 3067, 3002, 1610, 1521, 1461, 1352, 1251, 1170, 904 cm^{-1}.

^{1}H NMR (400 MHz, $CDCl_3$): δ = 8.25-8.23 (m, 1 H, Ar-H), 8.22 (t, J = 2.4 Hz, 1 H, Ar-H), 7.52 (s, 1 H, Ar-H), 7.03-7.02 (m, 1 H, Ar-H), 7.00 (t, J = 2.4 Hz, 1 H, Ar-H), 3.88 (s, 3 H, CH_3), 3.02 (s, 3 H, CH_3).

^{13}C NMR (100 MHz, $CDCl_3$): δ = 166.4, 162.1, 153.9, 151.0, 147.2, 129.6, 122.3, 114.4, 107.8, 55.5, 19.9.

HRMS (ESI): *m/z* $[M + H]^+$ *calcd* for $C_{13}H_{11}N_4ClO$: 275.0700; found: 275.0729.

7-Chloro-2-ethyl-5-methyl-[1,2,4]triazolo[1,5-*c*]pyrimidine (6j)

Yield: 0.067 g (34%); white powder; mp 142-144 °C.

IR (KBr): 3050, 2916, 1708, 1617, 1544, 1347, 1203, 1116, 900, 876 cm^{-1}.

^{1}H NMR (400 MHz, $CDCl_3$): δ = 7.48 (s, 1 H, Ar-H), 2.99-2.93 (m, 5 H, CH_2, CH_3), 1.42 (t, J = 7.6 Hz, 3 H, CH_3).

^{13}C NMR (100 MHz, $CDCl_3$): δ = 171.3, 153.5, 150.9, 147.1, 107.8, 22.4, 19.8, 12.4.

HRMS (ESI): *m/z* $[M + H]^+$ *calcd* for $C_8H_9N_4Cl$: 197.0594; found: 197.0601.

7-Chloro-2,5-diphenyl-[1,2,4]triazolo[1,5-*c*]pyrimidine (6k)

Yield: 0.26 g (84%); white powder; mp 173-174 °C.

IR (KBr): 3063, 2920, 1617, 1524, 1474, 1355, 1222, 1190, 1088, 982, 855 cm^{-1}.

^{1}H NMR (400 MHz, $CDCl_3$): δ = 8.78 (d, J = 7.2 Hz, 2 H, Ar-H), 8.35 (s, 2 H, Ar-H), 7.69-7.61 (m, 4 H, Ar-H), 7.54 (s, 3 H, Ar-H).

^{13}C NMR (100 MHz, $CDCl_3$): δ = 166.5, 155.7, 148.8, 147.7, 132.8, 131.2, 131.0, 130.3, 129.8, 128.9, 128.7, 128.1, 108.2.

HRMS (ESI): *m/z* $[M + H]^+$ *calcd* for $C_{17}H_{11}N_4Cl$: 307.0750; found: 307.0759.

7-Chloro-2-(2-chlorophenyl)-5-phenyl-[1,2,4]triazolo[1,5-*c*]pyrimidine (6l)

Yield: 0.30 g (87%); white powder; mp177-178 °C.

IR (KBr): 3055, 2911, 1617, 1593, 1568, 1467, 1347, 1226, 1087, 981, 841 cm^{-1}.

^{1}H NMR (400 MHz, $CDCl_3$): δ = 8.81 (d, J = 7.2 Hz, 2 H, Ar-H), 8.13-8.11 (m, 1 H, Ar-H), 7.69 (s, 1 H, Ar-H), 7.66-7.58 (m, 4 H, Ar-H), 7.47-7.41 (m, 2 H, Ar-H).

^{13}C NMR (100 MHz, $CDCl_3$): δ = 164.9, 154.8, 149.0, 147.8, 133.7, 132.9, 132.4, 131.5, 131.2, 131.0, 130.1, 128.9, 128.7, 127.0, 108.4.

HRMS (ESI): *m/z* $[M + H]^+$ *calcd* for $C_{17}H_{10}N_4Cl_2$: 341.0361; found: 341.0394.

7-Chloro-2-(2-furanyl)-5-phenyl-[1,2,4]triazolo[1,5-*c*]pyrimidine (6m)

Yield: 0.26 g (87%); yellow powder; mp 190-191 °C.

IR (KBr): 3059, 2922, 1618, 1521, 1465, 1360, 1223, 1186, 1089, 1021, 981 cm^{-1}.

^{1}H NMR (400 MHz, $CDCl_3$): δ = 8.70 (d, *J* = 7.6 Hz, 2 H, Ar-H), 7.65 (s, 1 H, Ar-H), 7.63-7.58 (m, 4 H, Ar-H), 7.29 (d, *J* = 3.2 Hz, 1 H, Ar-H), 6.60 (s, 1 H, Ar-H).

^{13}C NMR (100 MHz, $CDCl_3$): δ = 159.0, 155.5, 149.0, 148.1, 145.3, 132.8, 130.9, 130.1, 128.7, 113.7, 112.2, 108.1.

HRMS (ESI): *m/z* $[M + H]^+$ *calcd* for $C_{15}H_9N_4ClO$: 297.0543; found: 297.0564.

7-Chloro-2-(4-methoxyphenyl)-5-phenyl-[1,2,4]triazolo[1,5-*c*]pyrimidine (6n)

Yield: 0.24 g (71%); white powder; mp 174-175 °C.

IR (KBr): 2930, 2834, 1727, 1612, 1573, 1458, 1352, 1246, 1174, 1039, 981 cm^{-1}.

^{1}H NMR (400 MHz, $CDCl_3$): δ = 8.74 (d, *J* = 8.0 Hz, 2 H, Ar-H), 8.23 (d, *J* = 8.8 Hz, 2 H, Ar-H), 7.64-7.57 (m, 3 H, Ar-H), 7.55 (s, 1 H, Ar-H), 6.99 (d, *J* = 8.4 Hz, 2 H, Ar-H), 3.87 (s, 3 H, CH_3).

^{13}C NMR (100 MHz, $CDCl_3$): δ = 166.3, 162.1, 155.6, 148.5, 147.5, 132.7, 130.9, 130.3, 129.6, 128.6, 122.2, 114.3, 107.9, 55.5.

HRMS (ESI): *m/z* $[M + H]^+$ *calcd* for $C_{18}H_{13}N_4ClO$: 337.0856; found: 337.0863.

7-Chloro-2-ethyl-5-phenyl-[1,2,4]triazolo[1,5-*c*]pyrimidine (6o)

Yield: 0.093 g (36%); white powder; mp 157-158 °C.

IR (KBr): 3045, 2922, 1730, 1612, 1520, 1473, 1448, 1350, 1222, 1088, 867 cm^{-1}.

^{1}H NMR (400 MHz, $CDCl_3$): δ = 8.67 (d, J = 7.2 Hz, 2 H, Ar-H), 7.64-7.56 (m, 4 H, Ar-H), 3.01 (q, J = 7.2 Hz, 2 H, CH_2), 1.46 (t, J = 7.2 Hz, 3 H, CH_3).

^{13}C NMR (100 MHz, $CDCl_3$): δ = 171.5, 155.3, 148.7, 147.5, 132.7, 130.8, 130.3, 128.6, 107.9, 22.5, 12.4.

HRMS (ESI): *m/z calcd* for $C_{13}H_{11}N_4Cl$ $[M + H]^+$ 259.0750; found: 259.0767.

References

1. Sharefkin, J. G.; Saltzman, H. *Org. Synth. Coll. Vol. V* **1973**, 660–663.
2. Li, C.; Li, Z.; Wang, Q. *Synlett* **2010**, *21*, 2179–2183.
3. Kenner, G. W.; Lythgoe, B.; Todd, A. R.; Topham, A. *J. Chem. Soc.* **1943**, 388–390.
