# Supplementary material for: IBD-mediated oxidative cyclization of pyrimidinylhydrazones and concurrent Dimroth rearrangement: Synthesis of [1,2,4]triazolo[1,5-c]pyrimidine derivatives
Source: Beilstein J Org Chem. 2013 Nov 25;9:2629–34. doi: 10.3762/bjoc.9.298 (PMC3869367; doi:10.3762/bjoc.9.298)

Supporting Information

for

IBD-mediated oxidative cyclization of pyrimidinylhydrazones and concurrent Dimroth rearrangement: Synthesis of [1,2,4]triazolo[1,5-*c*]pyrimidine derivatives

Caifei Tang, Zhiming Li and Quanrui Wang

Address: Department of Chemistry, Fudan University, 220 Handan Road, 200433 Shanghai, P. R. China

Email: Quanrui Wang* - qrwang@fudan.edu.cn

*Corresponding author

NMR spectral data for unknown compounds

Spectra of (6-chloro-4-pyrimidinyl)hydrazones **4**……………………………………… S2

Spectra of 7-chloro-5-methyl-3-phenyl[1,2,4]triazolo[4,3-*c*]pyrimidine (**5f**)…………S15

Spectra of 7-chloro-[1,2,4]triazolo[1,5-*c*]pyrimidines **6**………… ………………… S16

Spectra of (6-chloro-4-pyrimidinyl)hydrazones 4

Benzaldehyde (6-chloro-4-pyrimidinyl)hydrazone (4a)

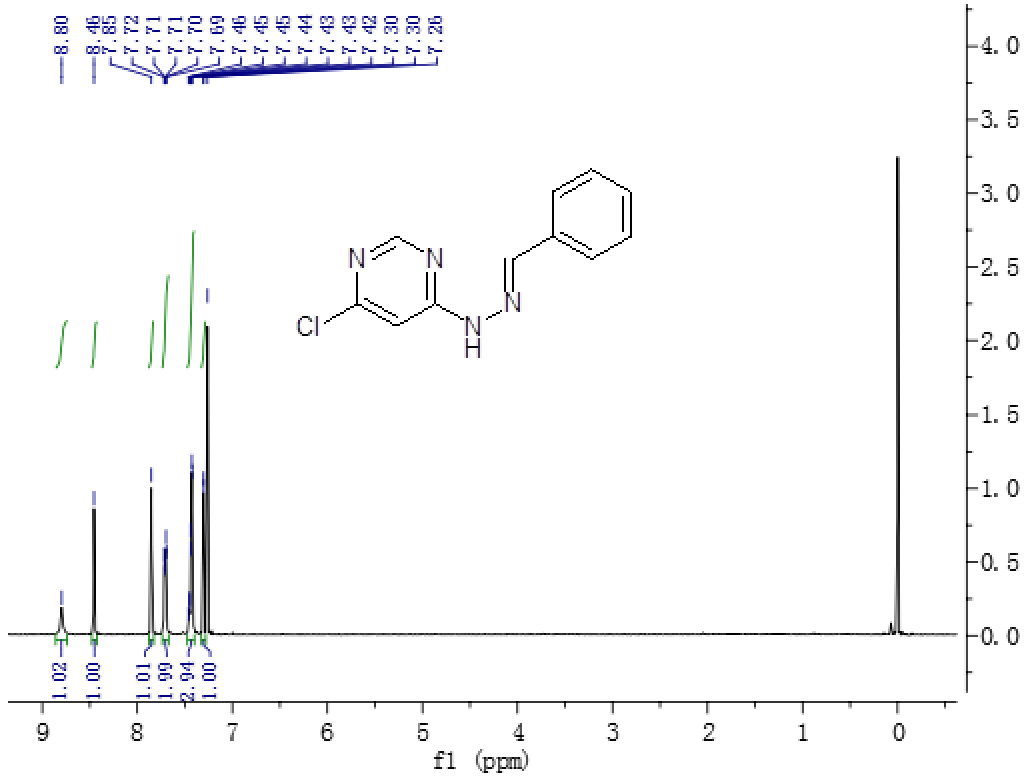

2-Chlorobenzaldehyde (6-chloro-4-pyrimidinyl)hydrazone (4b)

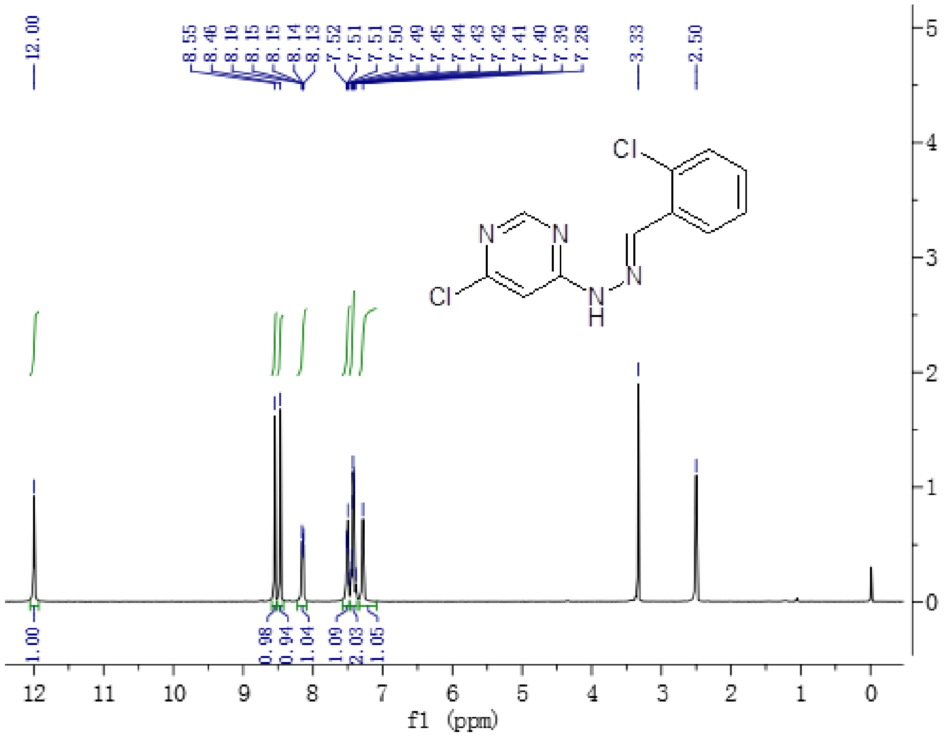

Furfural (6-chloro-4-pyrimidinyl)hydrazone (4c)

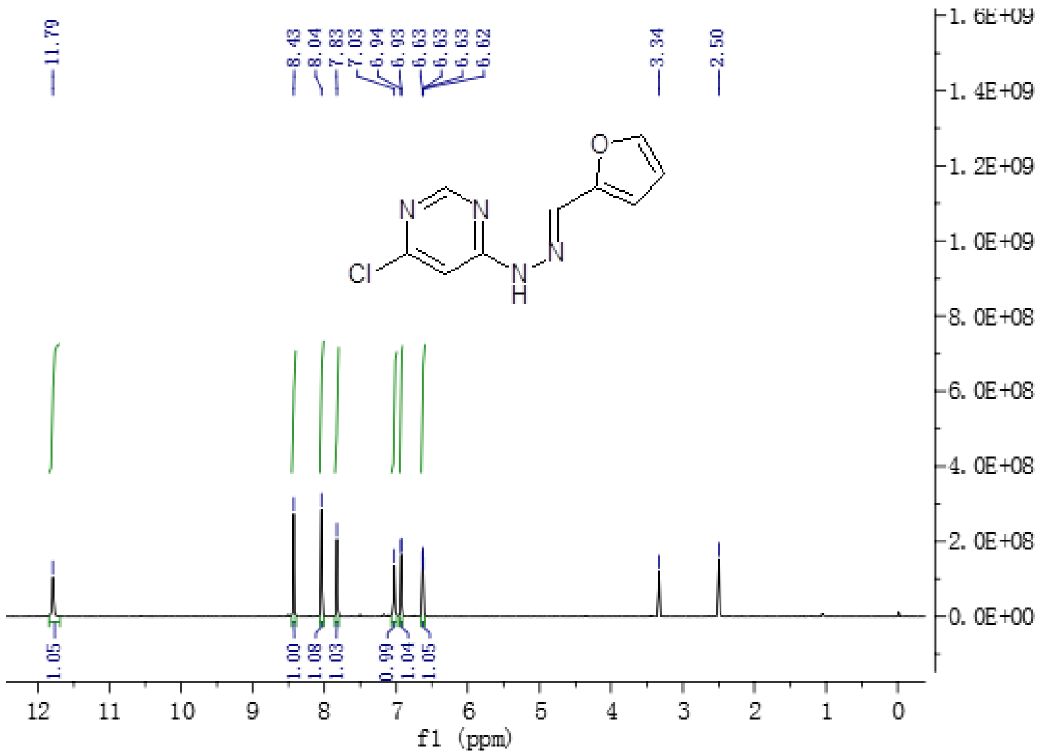

Furfural (6-chloro-4-pyrimidinyl)hydrazone (4c)

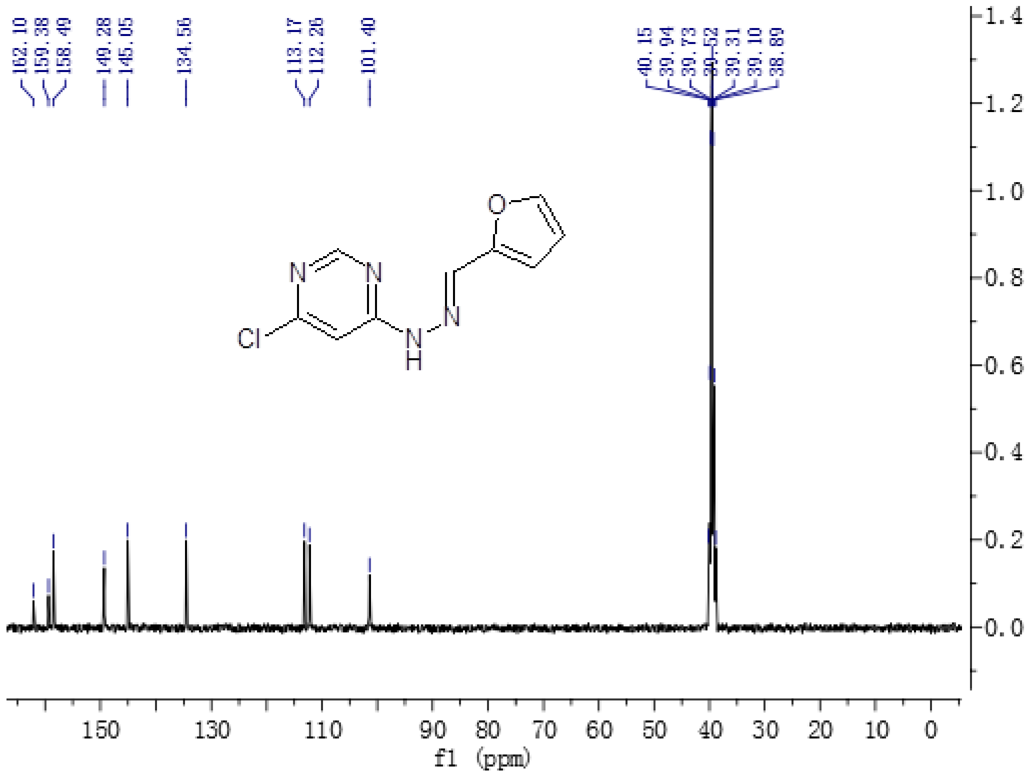

4-Methoxybenzaldehyde (6-chloro-4-pyrimidinyl)hydrazone (4d)

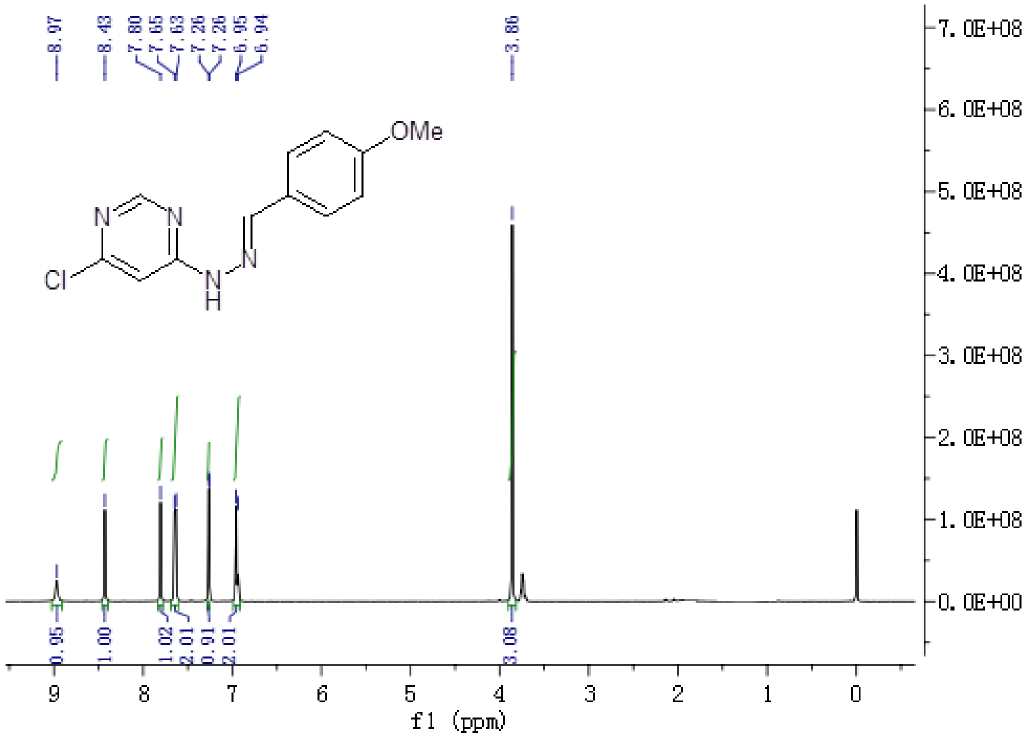

Propanaldehyde (6-chloro-4-pyrimidinyl)hydrazone (4e)

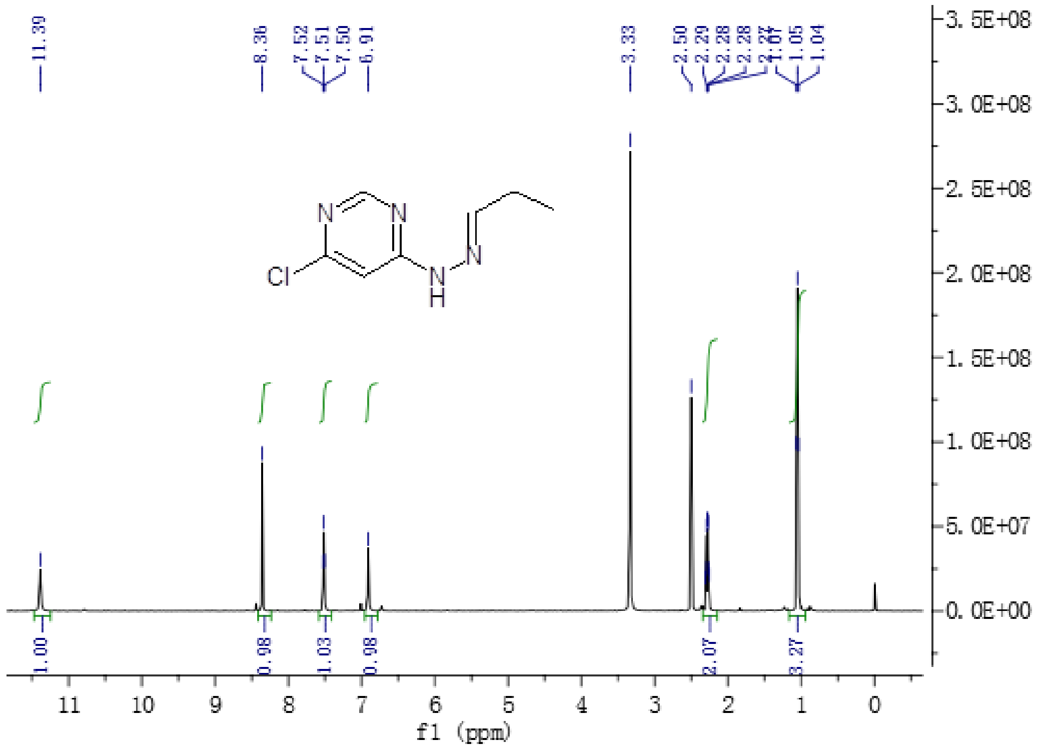

Benzaldehyde (6-chloro-2-methyl-4-pyrimidinyl)hydrazone (4f)

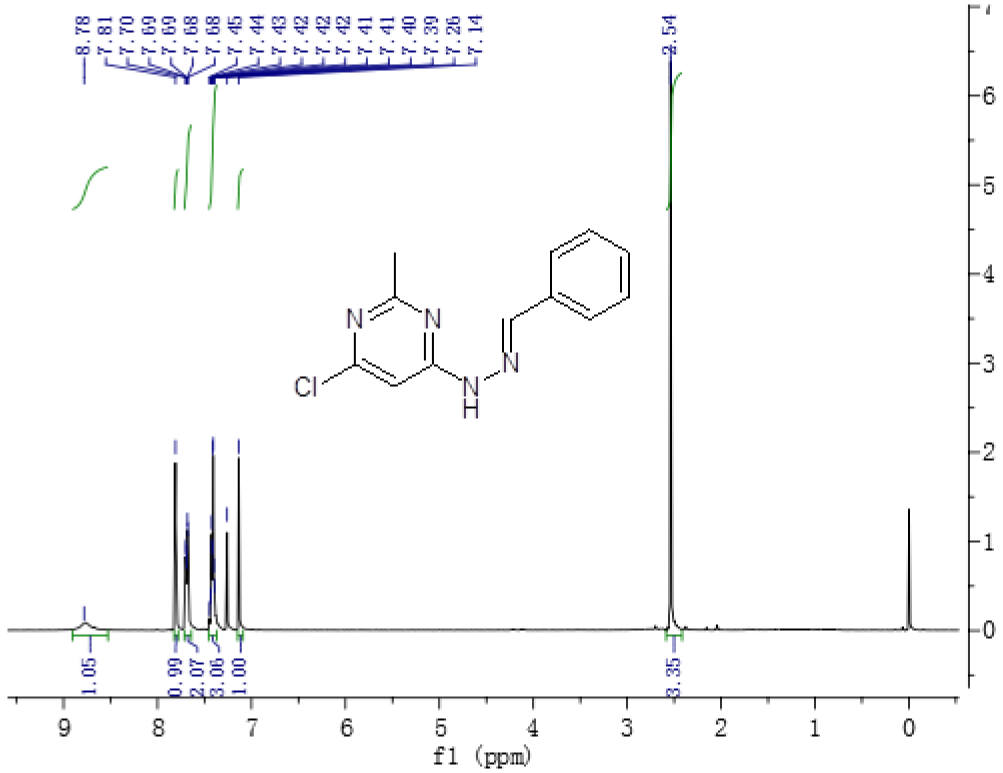

Benzaldehyde (6-chloro-2-methyl-4-pyrimidinyl)hydrazone (4f)

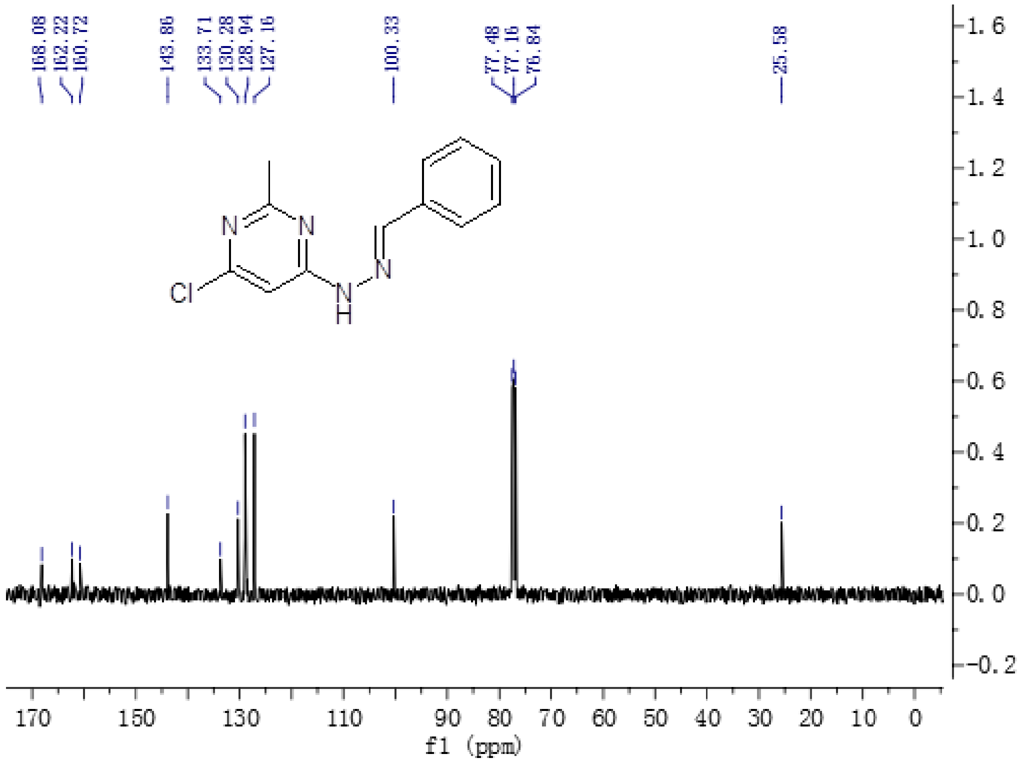

2-Chlorobenzaldehyde (6-chloro-2-methyl-4-pyrimidinyl)hydrazone (4g)

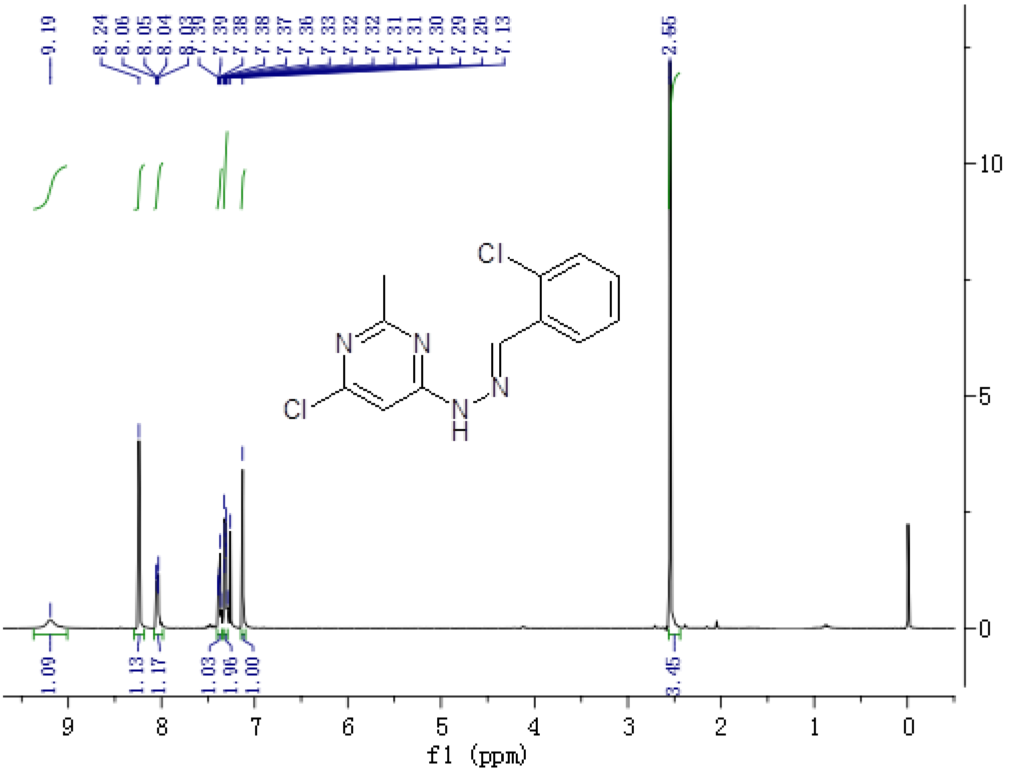

2-Chlorobenzaldehyde (6-chloro-2-methyl-4-pyrimidinyl)hydrazone (4g)

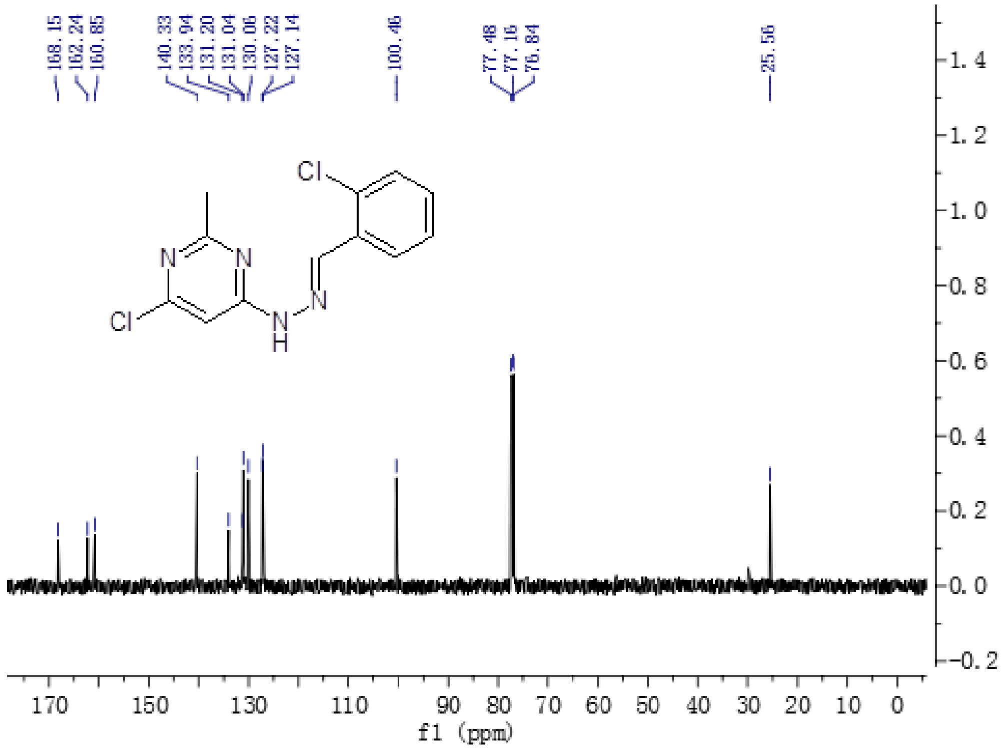

Furfural (6-chloro-2-methyl-4-pyrimidinyl)hydrazone (4h)

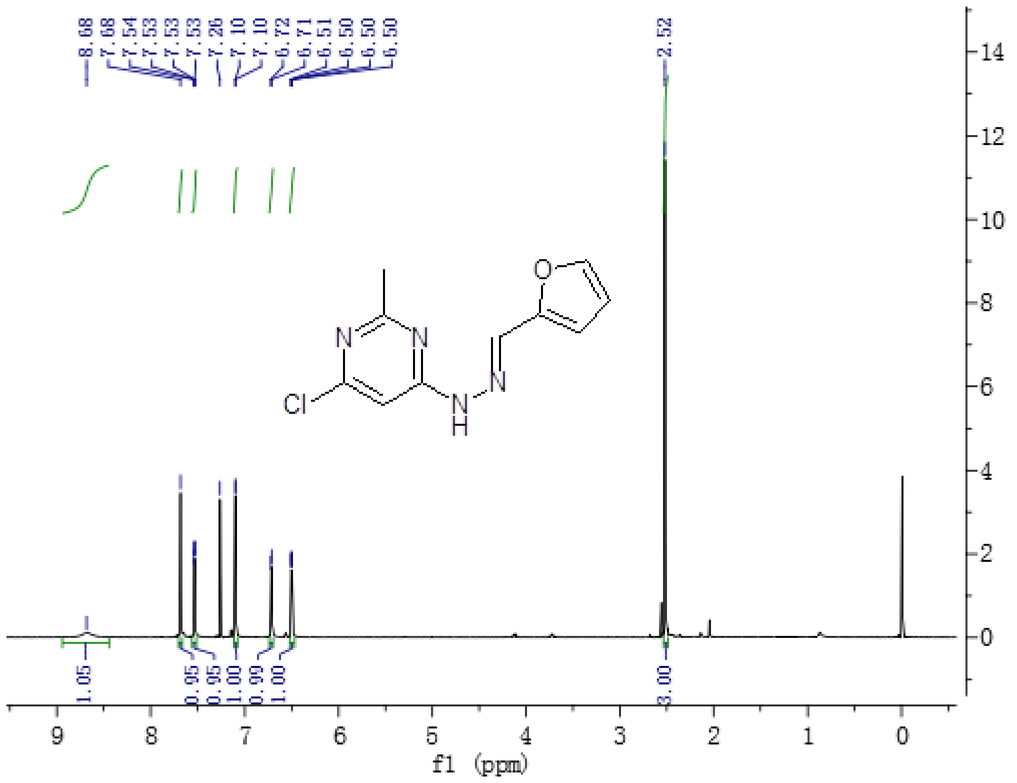

Furfural (6-chloro-2-methyl-4-pyrimidinyl)hydrazone (4h)

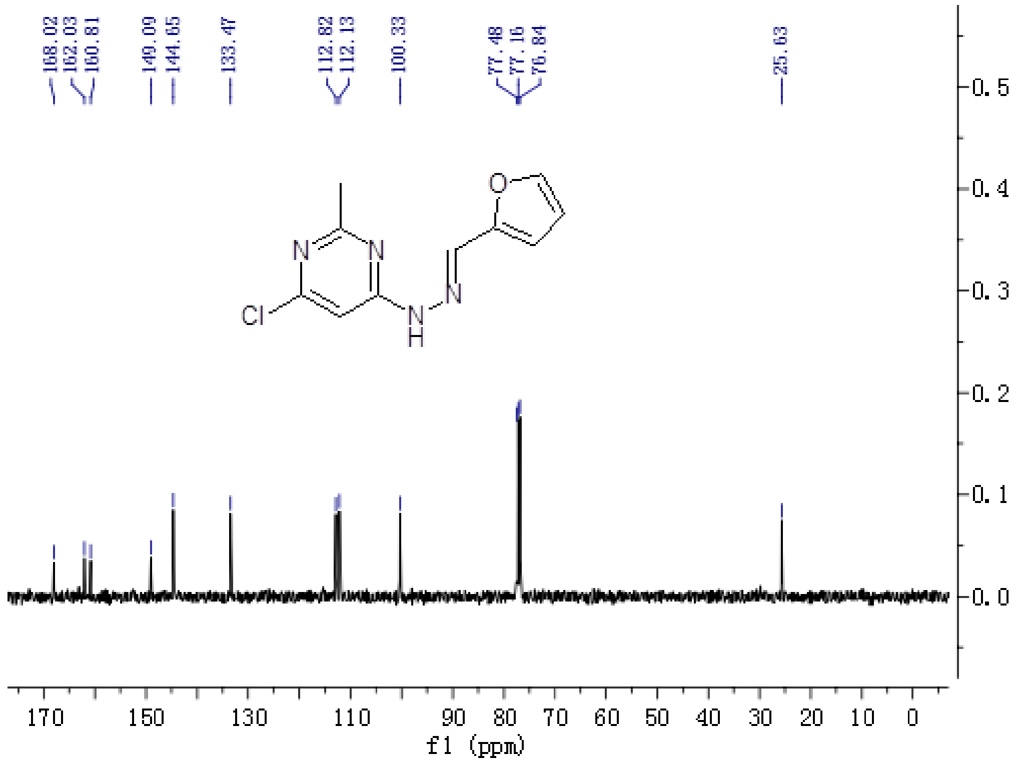

4-Methoxybenzaldehyde (6-chloro-2-methyl-4-pyrimidinyl)hydrazone (4i)

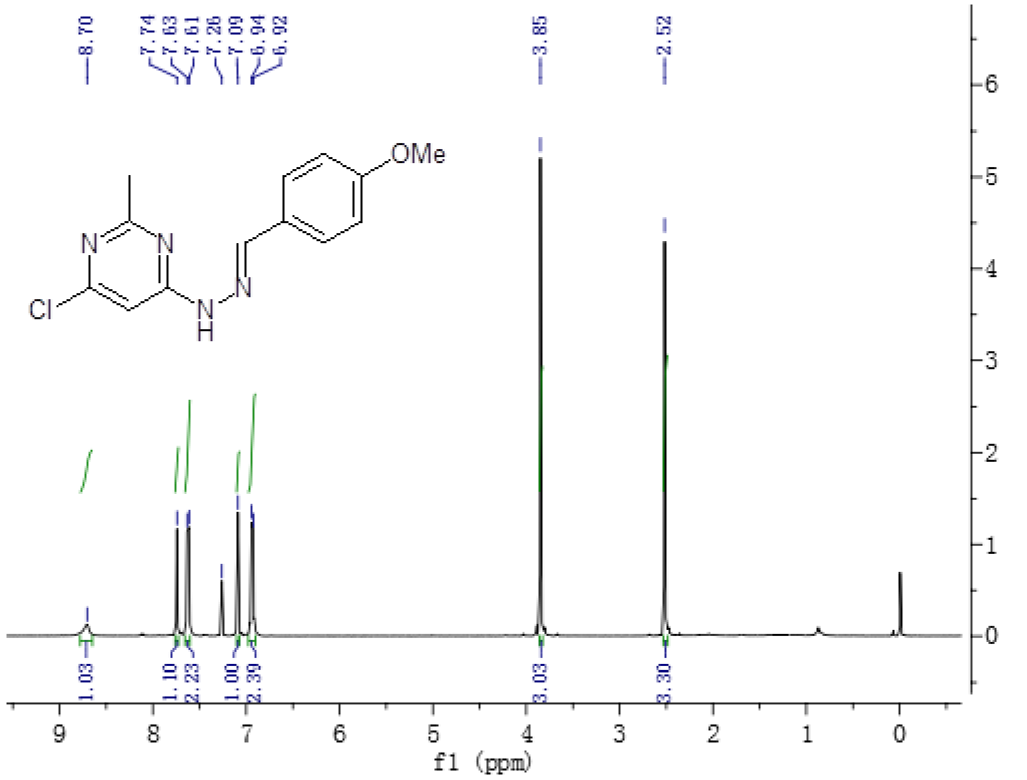

4-Methoxybenzaldehyde (6-chloro-2-methyl-4-pyrimidinyl)hydrazone (4i)

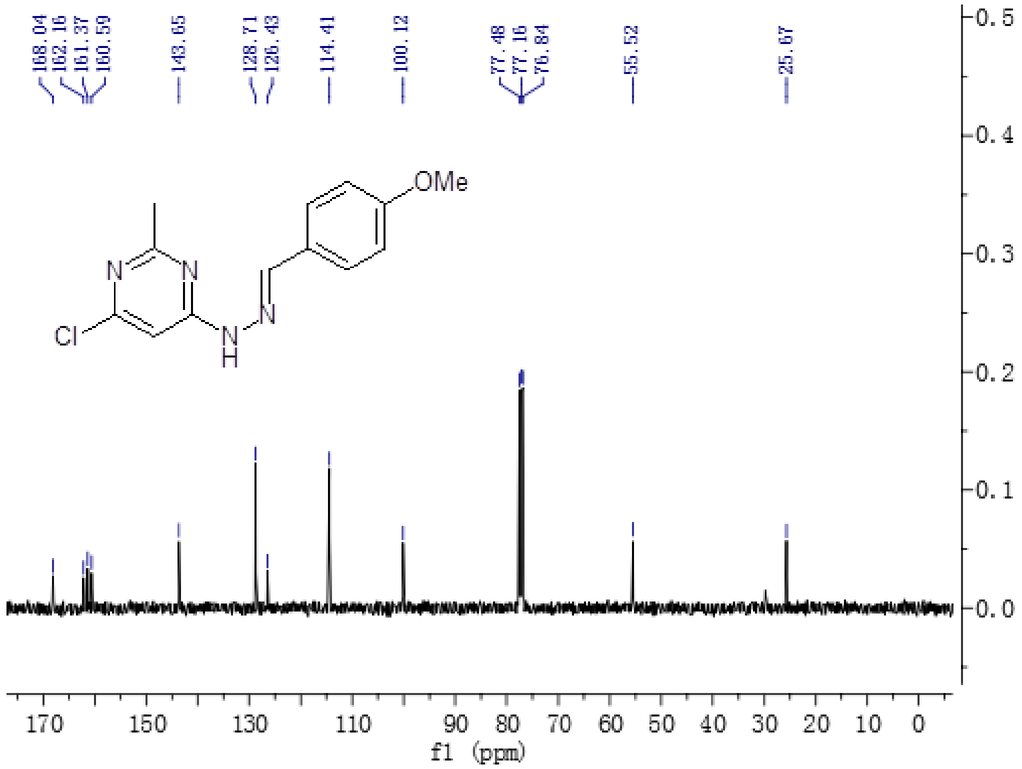

Propanaldehyde (6-chloro-2-methyl-4-pyrimidinyl)hydrazone (4j)

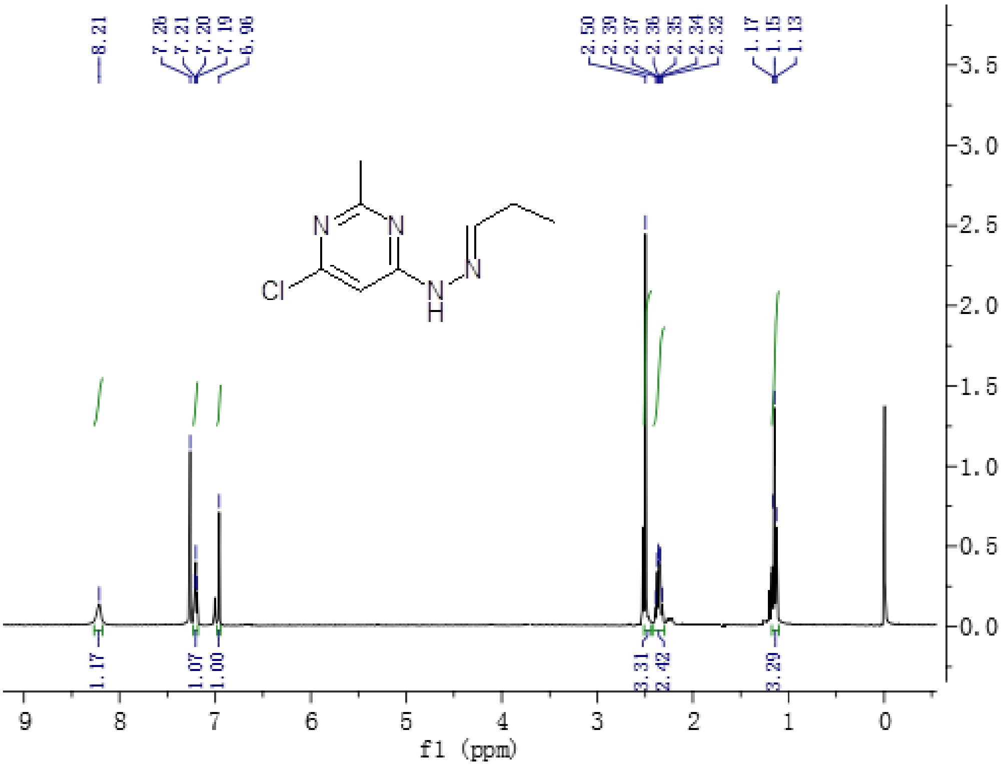

Propanaldehyde (6-chloro-2-methyl-4-pyrimidinyl)hydrazone (4j)

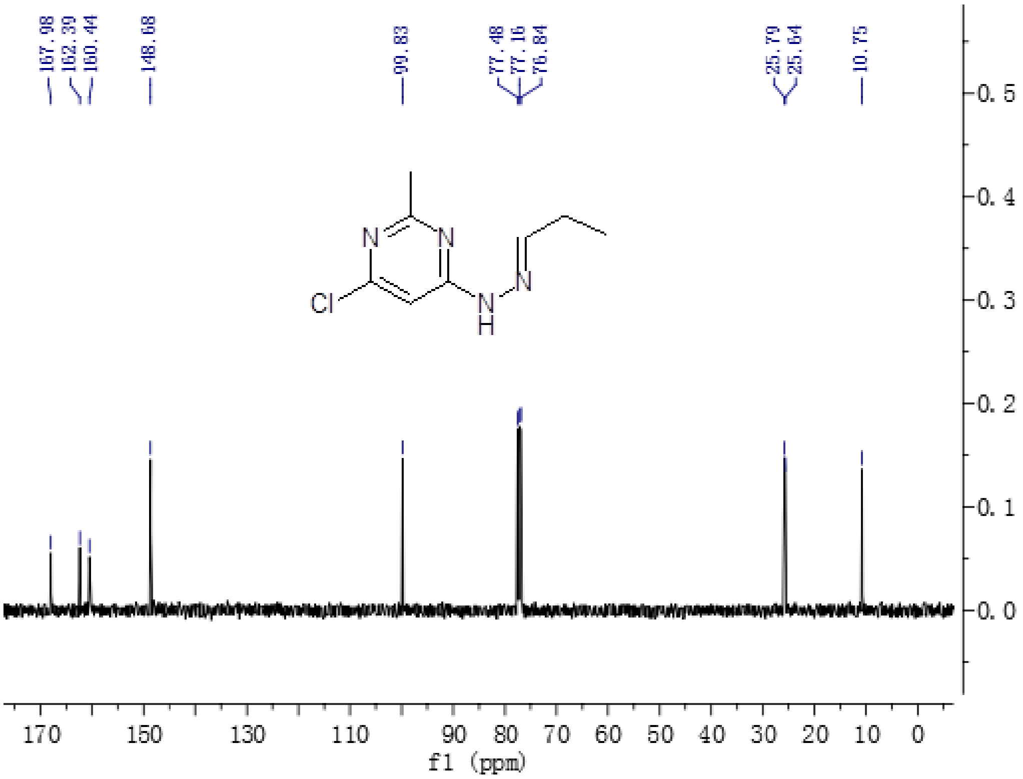

Benzaldehyde (6-chloro-2-phenyl-4-pyrimidinyl)hydrazone (4k)

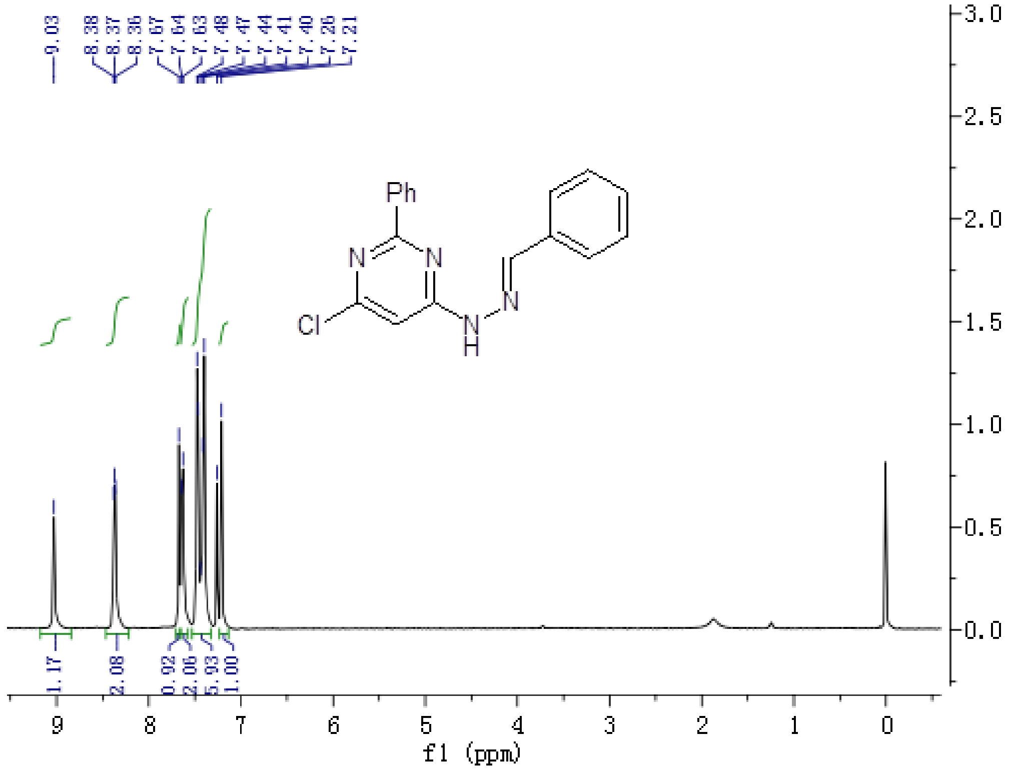

Benzaldehyde (6-chloro-2-phenyl-4-pyrimidinyl)hydrazone (4k)

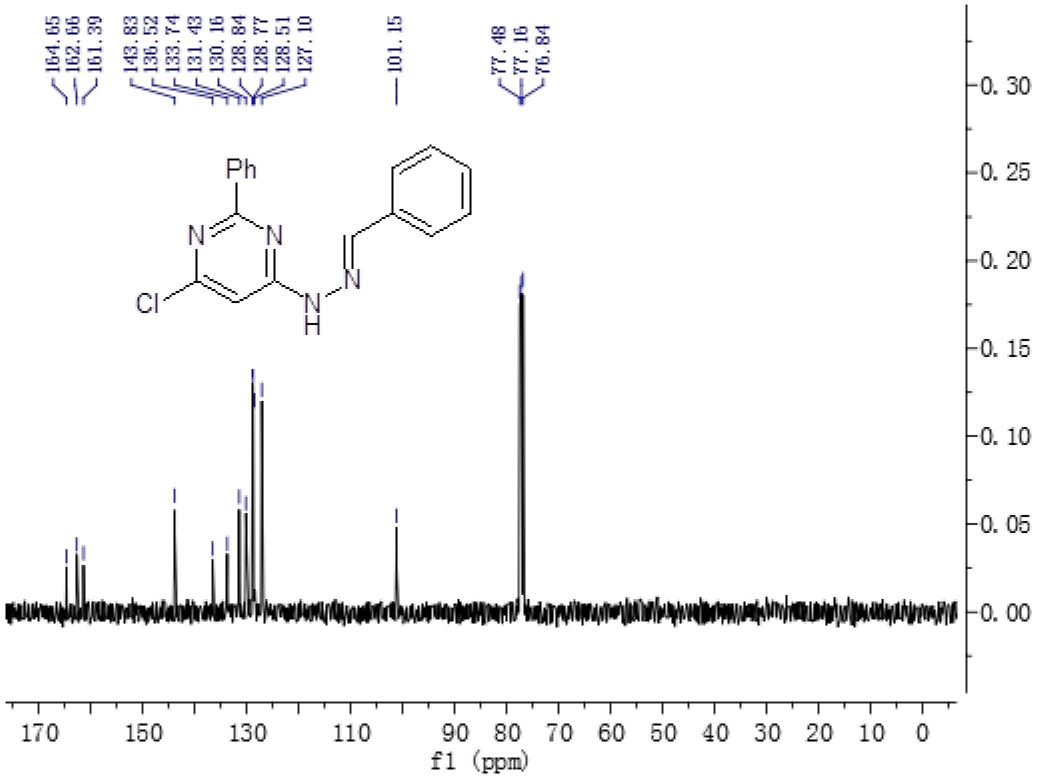

2-Chlorobenzaldehyde (6-chloro-2-phenyl-4-pyrimidinyl)hydrazone (4l)

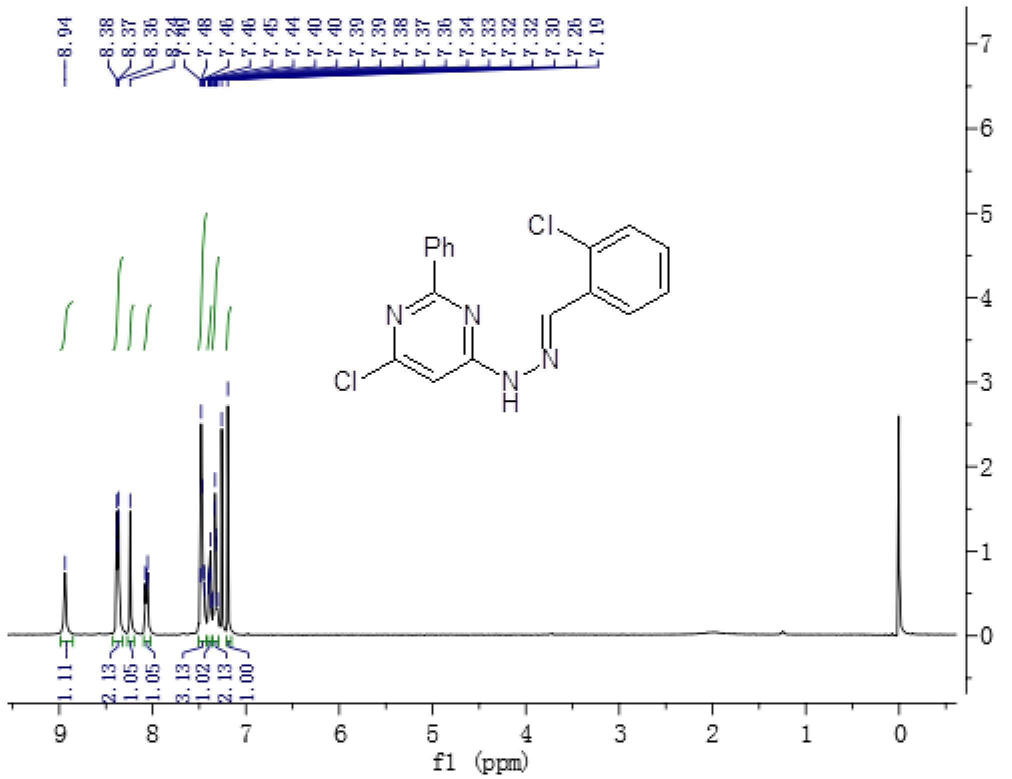

2-Chlorobenzaldehyde (6-chloro-2-phenyl-4-pyrimidinyl)hydrazone (4l)

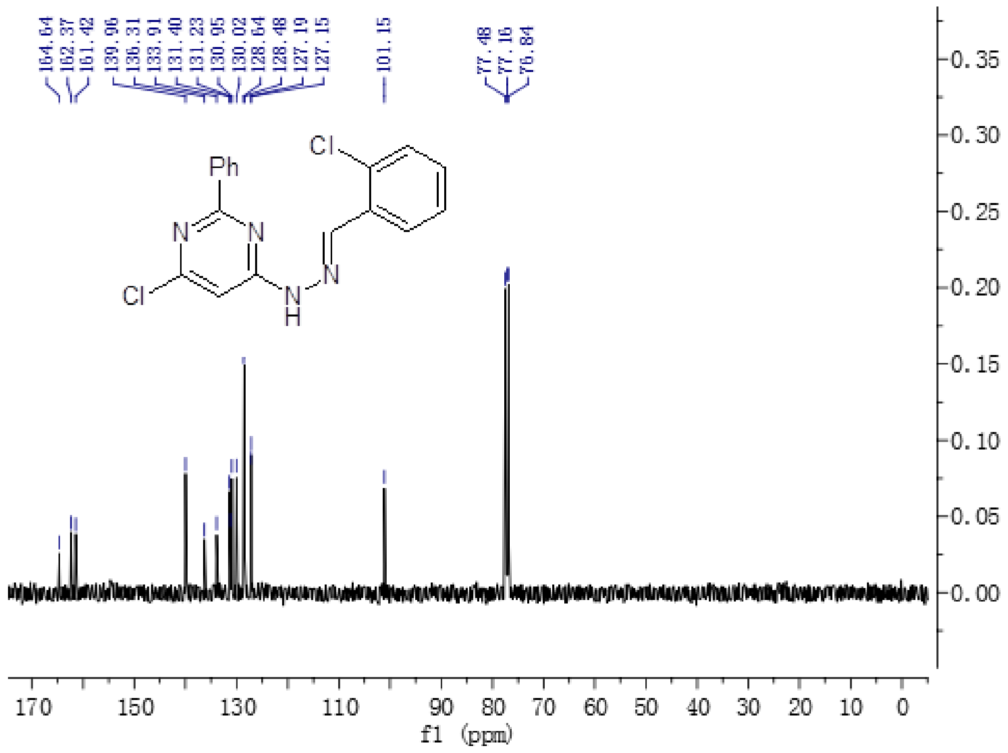

Furfural (6-chloro-2-phenyl-4-pyrimidinyl)hydrazone (4m)

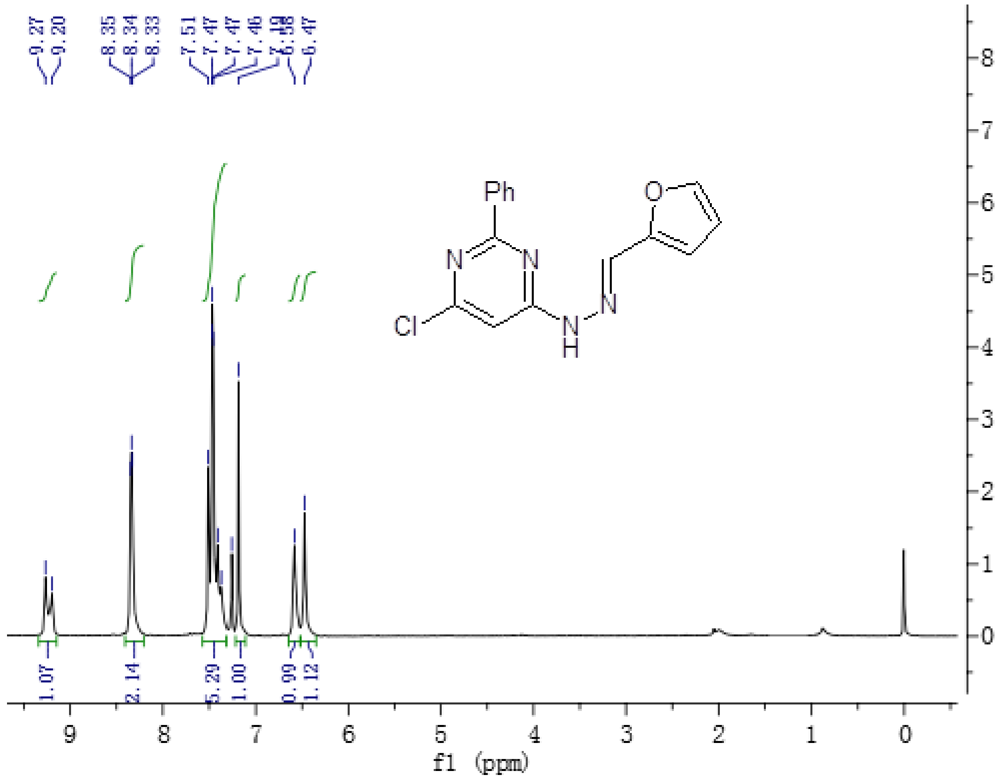

Furfural (6-chloro-2-phenyl-4-pyrimidinyl)hydrazone (4m)

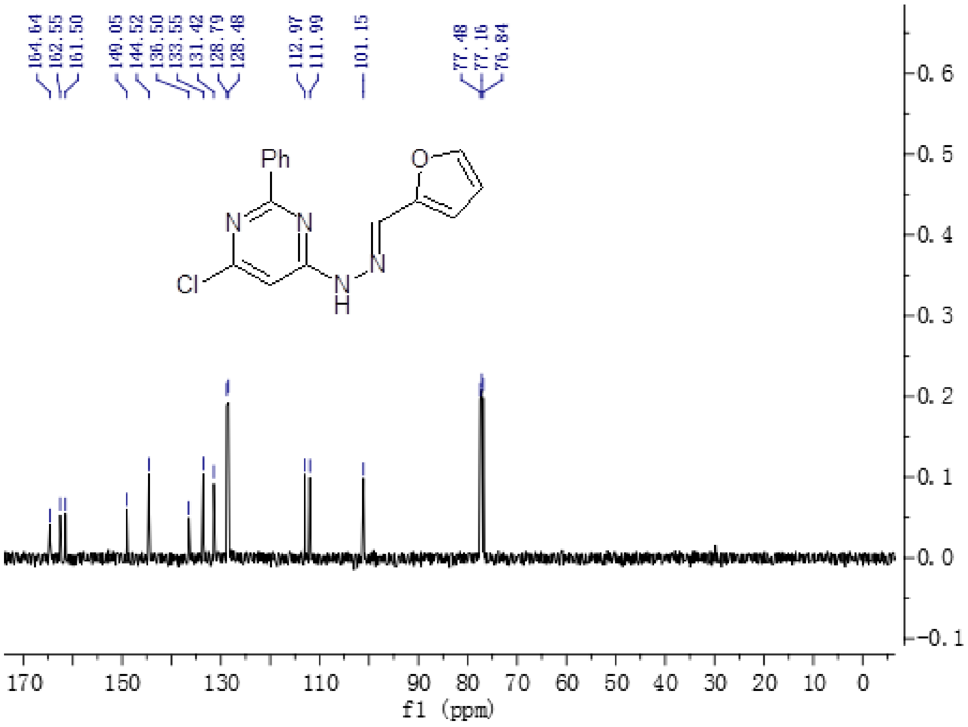

4-Methoxybenzaldehyde (6-chloro-2-phenyl-4-pyrimidinyl)hydrazone (4n)

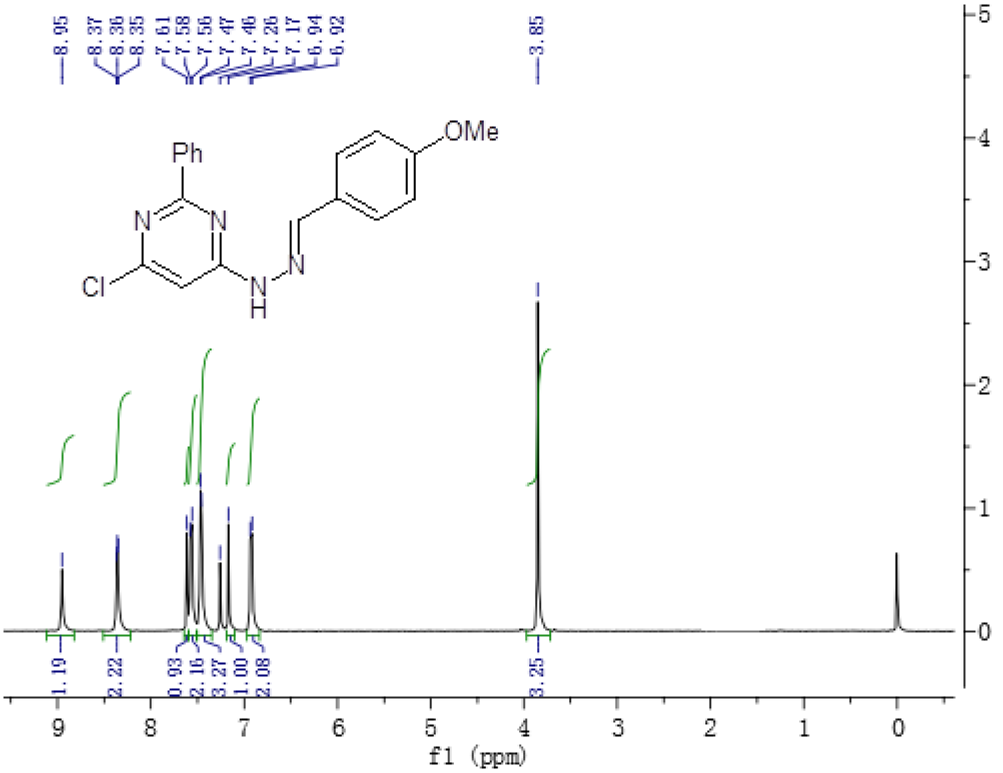

4-Methoxybenzaldehyde (6-chloro-2-phenyl-4-pyrimidinyl)hydrazone (4n)

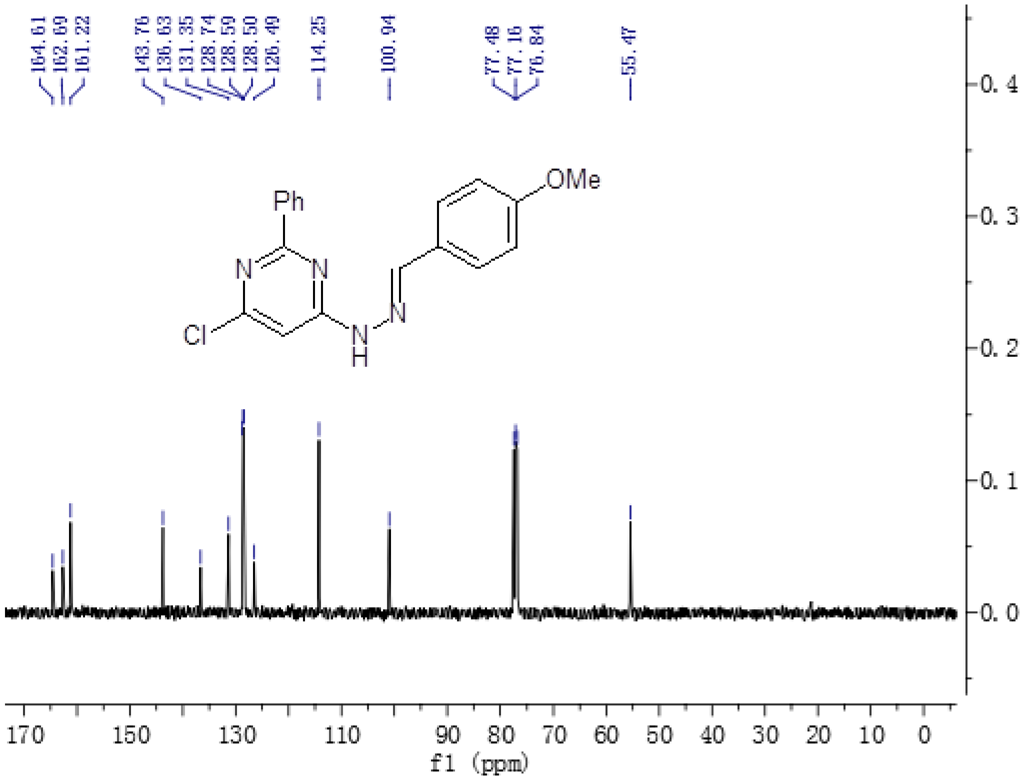

Propanaldehyde (6-chloro-2-phenyl-4-pyrimidinyl)hydrazone (4o)

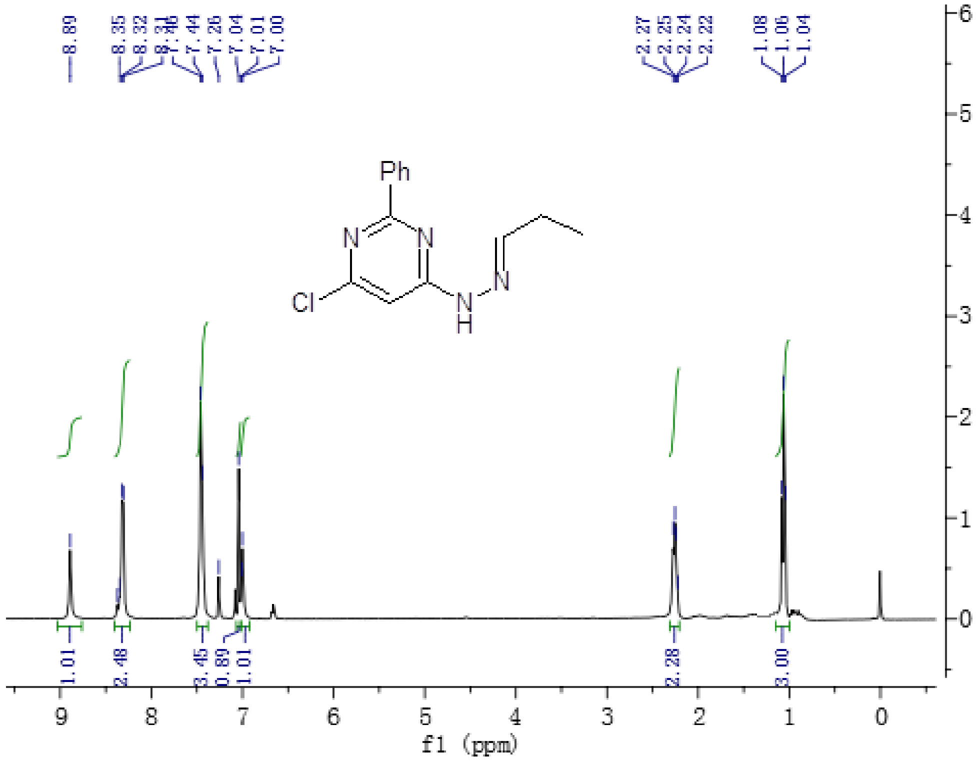

Propanaldehyde (6-chloro-2-phenyl-4-pyrimidinyl)hydrazone (4o)

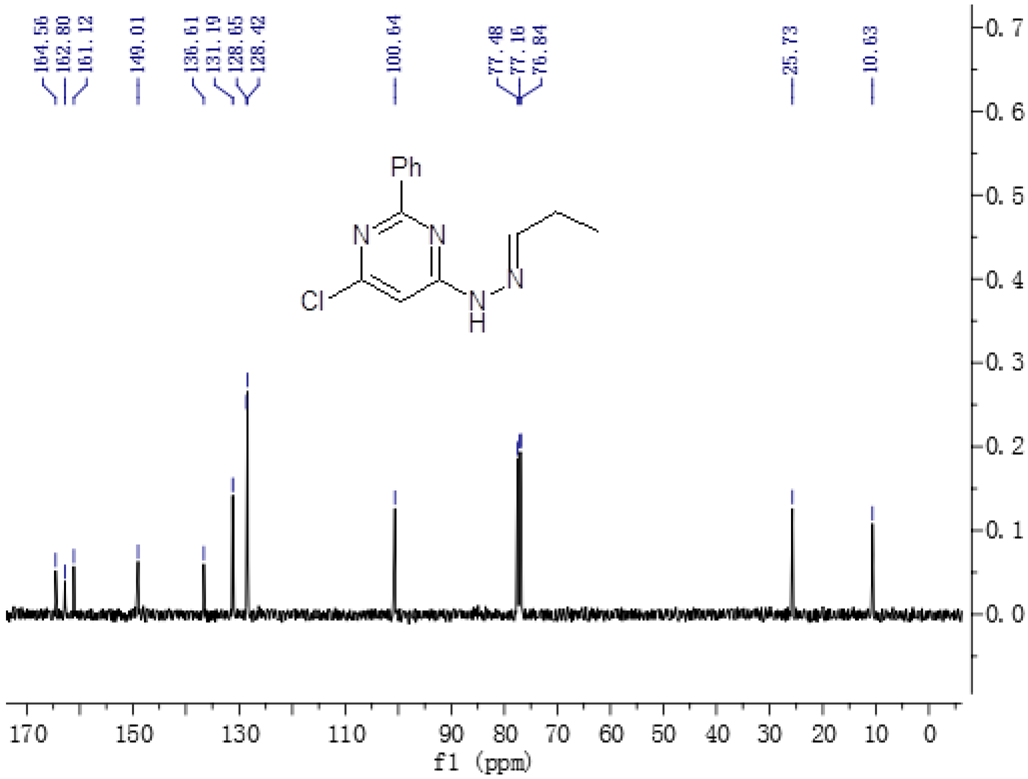

Spectra of 7-chloro-5-methyl-3-phenyl-[1,2,4]triazolo[4,3-*c*]pyrimidine (5f)

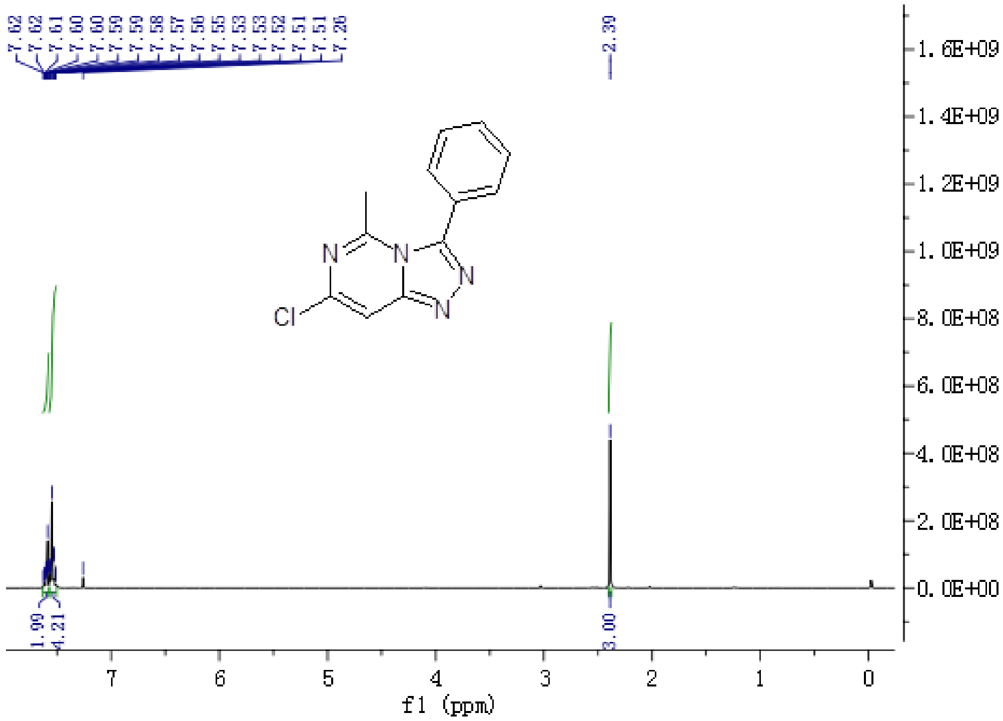

7-Chloro-5-methyl-3-phenyl-[1,2,4]triazolo[4,3-*c*]pyrimidine (5f)

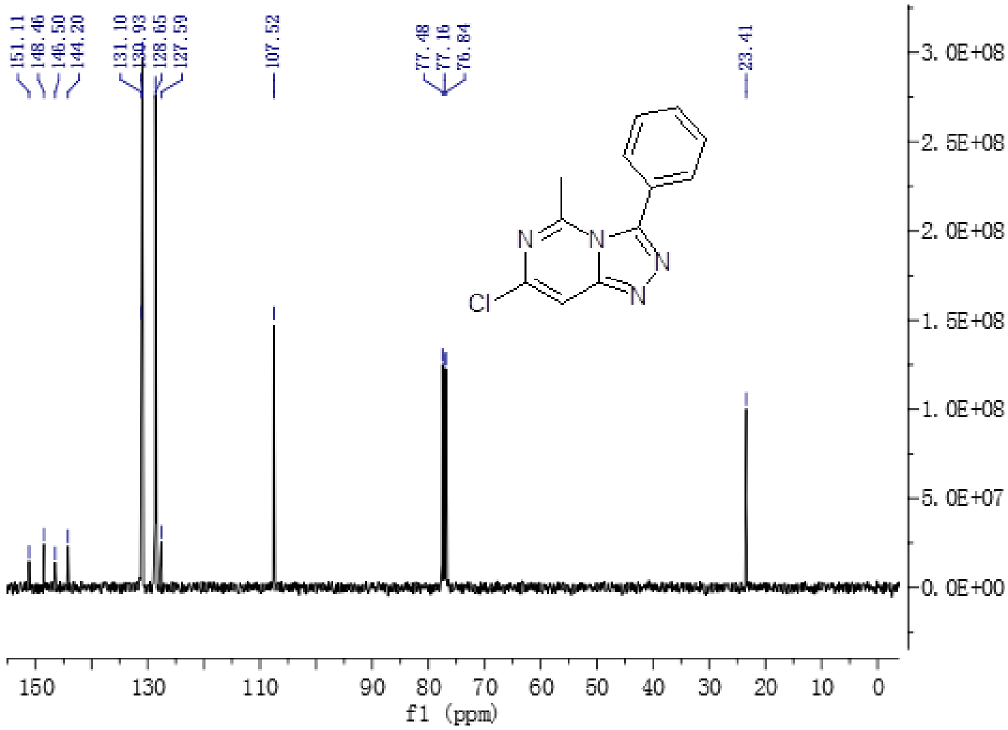

Spectra of 7-chloro-[1,2,4]triazolo[1,5-*c*]pyrimidines 6

7-Chloro-2-phenyl-[1,2,4]triazolo[1,5-*c*]pyrimidine (6a)

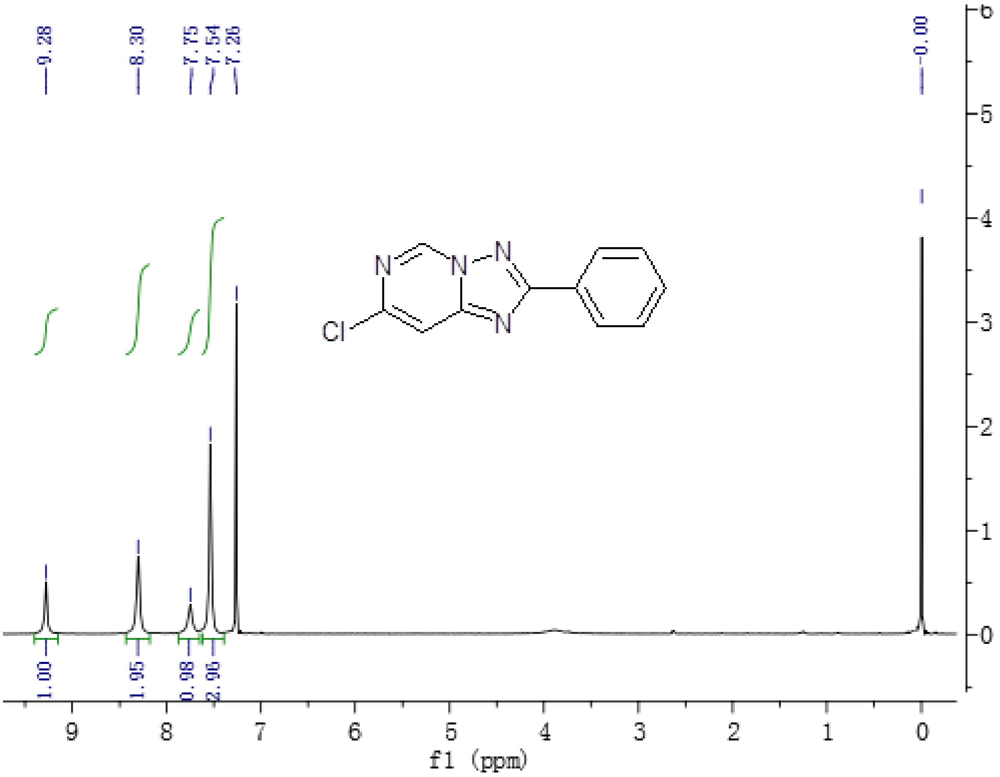

7-Chloro-2-phenyl-[1,2,4]triazolo[1,5-*c*]pyrimidine (6a)

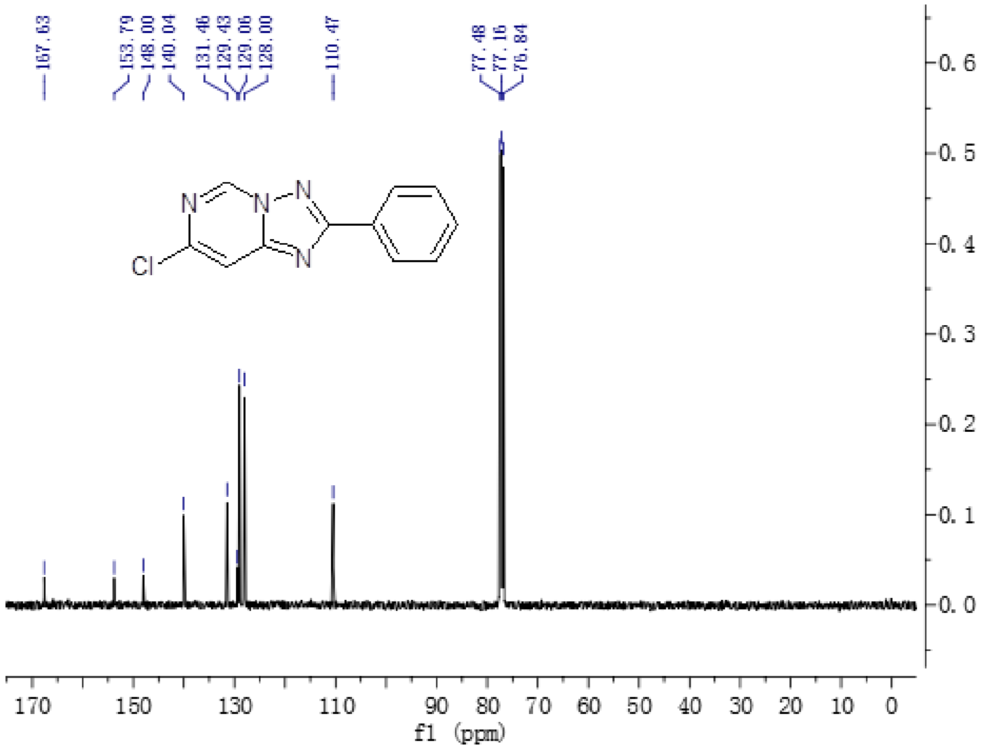

7-Chloro-2-(2-chlorophenyl)-[1,2,4]triazolo[1,5-*c*]pyrimidine (6b)

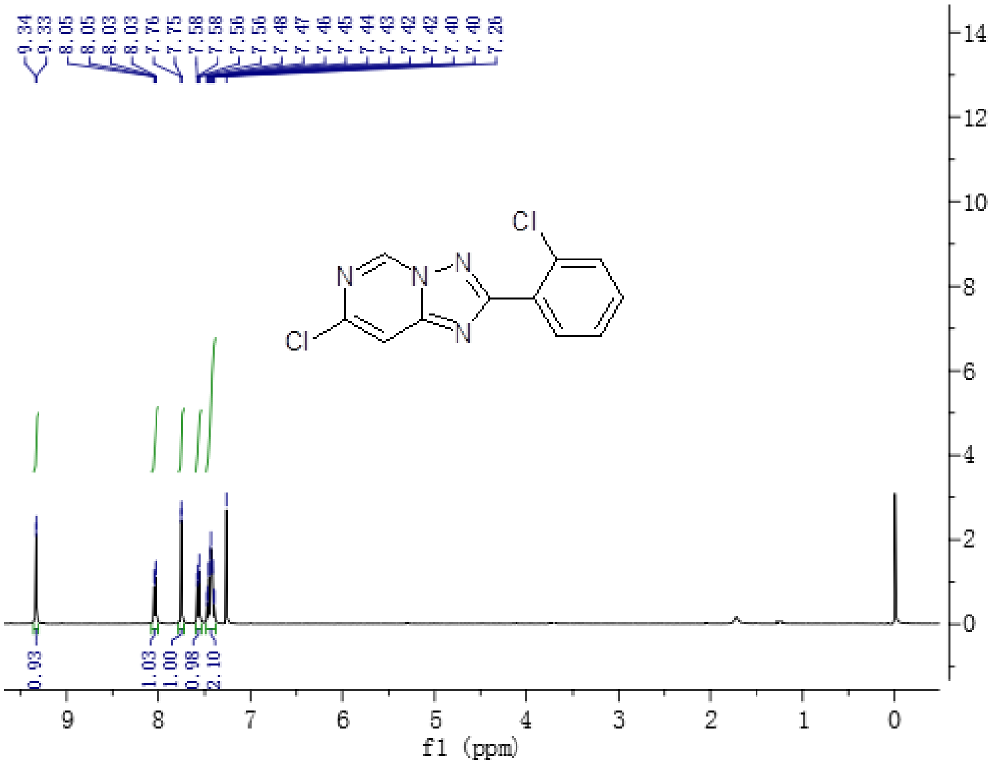

7-Chloro-2-(2-chlorophenyl)-[1,2,4]triazolo[1,5-*c*]pyrimidine (6b)

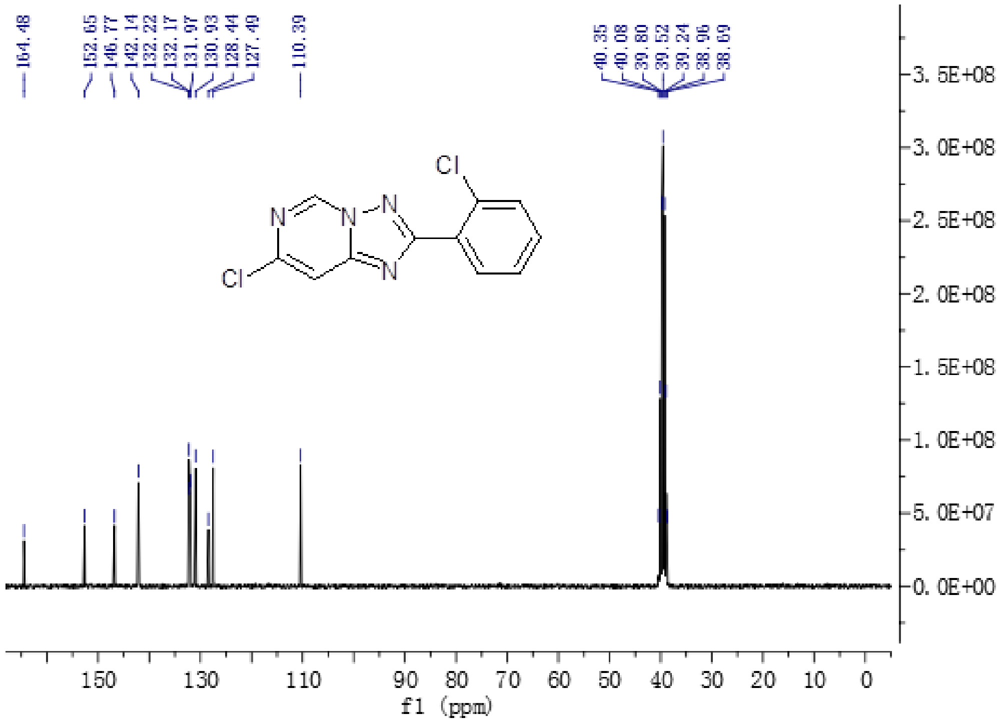

7-Chloro-2-(2-furanyl)-[1,2,4]triazolo[1,5-*c*]pyrimidine (6c)

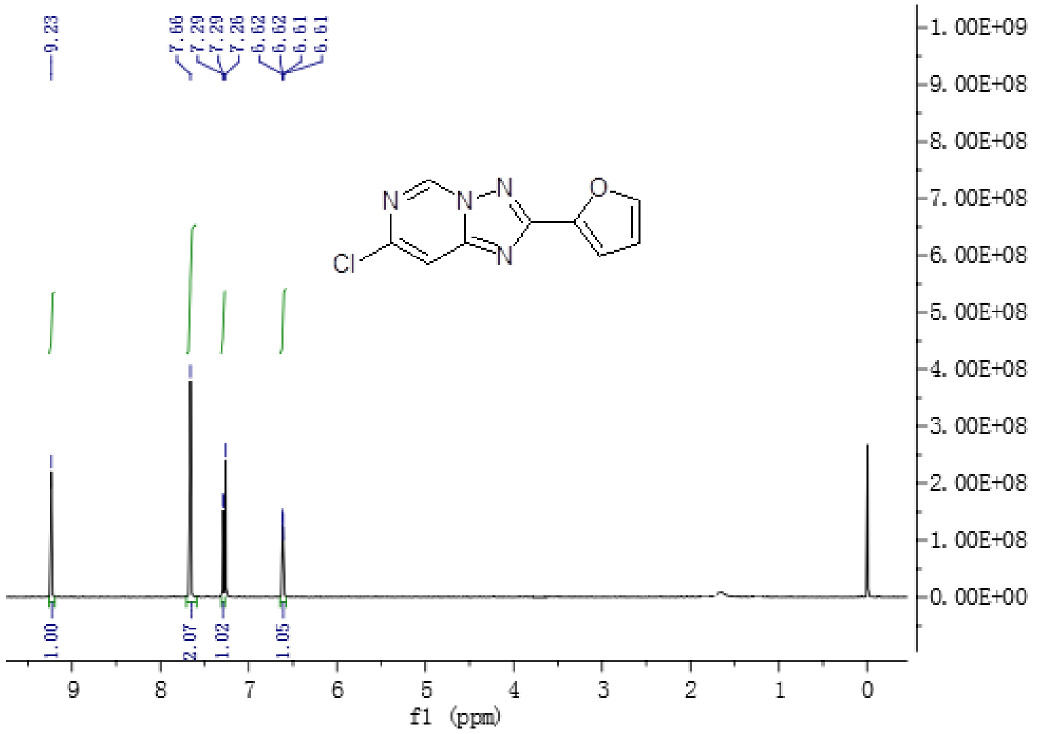

7-Chloro-2-(2-furanyl)-[1,2,4]triazolo[1,5-*c*]pyrimidine (6c)

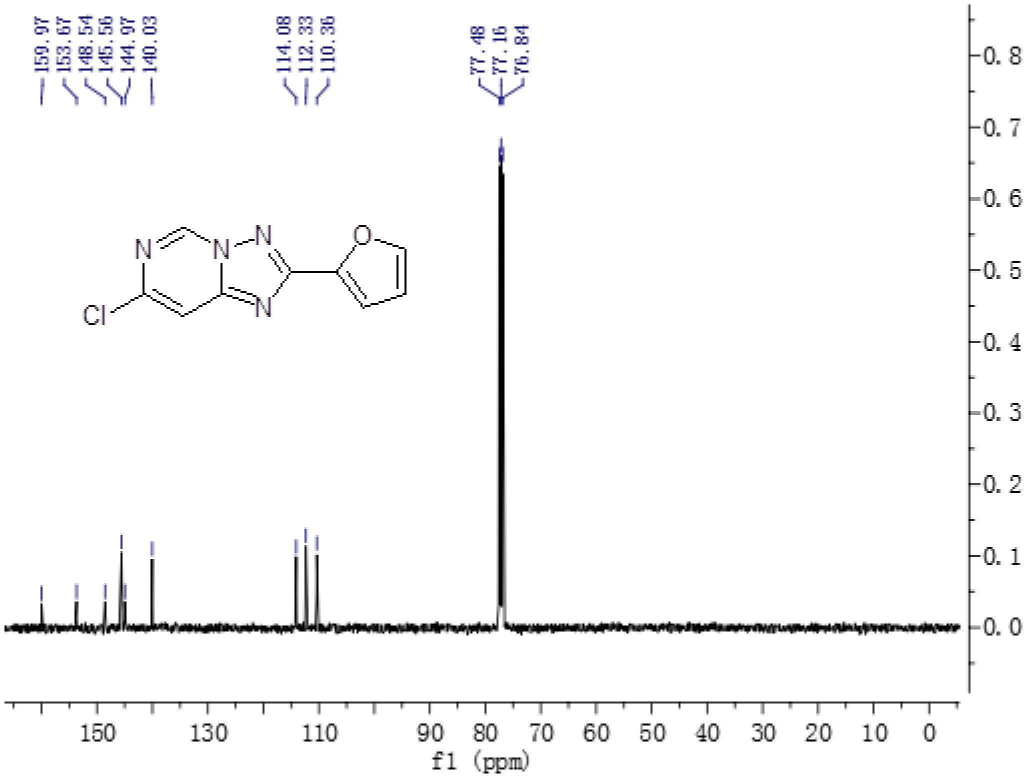

7-Chloro-2-(4-methoxyphenyl)-[1,2,4]triazolo[1,5-*c*]pyrimidine (6d)

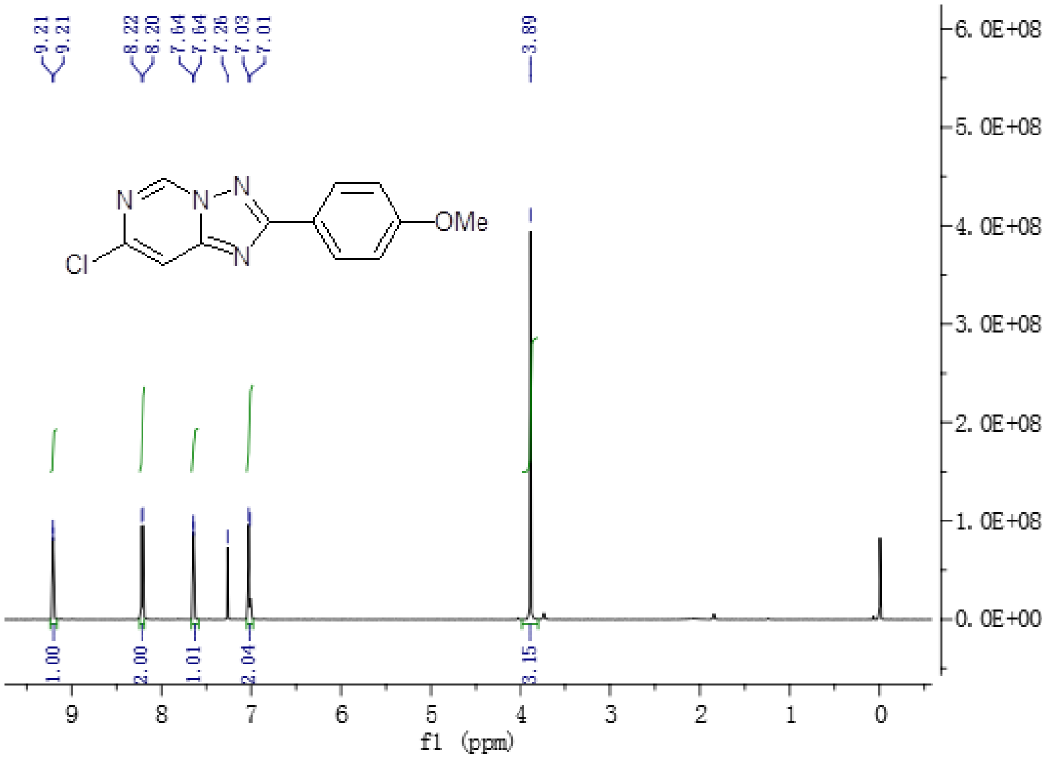

7-Chloro-2-(4-methoxyphenyl)-[1,2,4]triazolo[1,5-*c*]pyrimidine (6d)

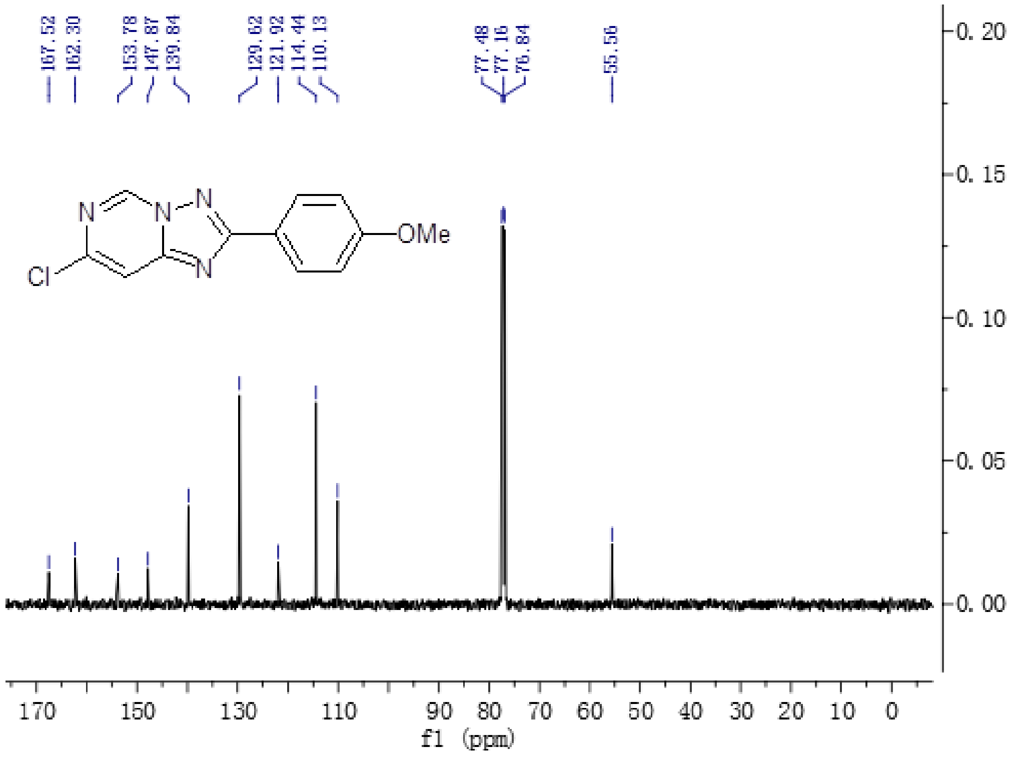

7-Chloro-2-ethyl-[1,2,4]triazolo[1,5-*c*]pyrimidine (6e)

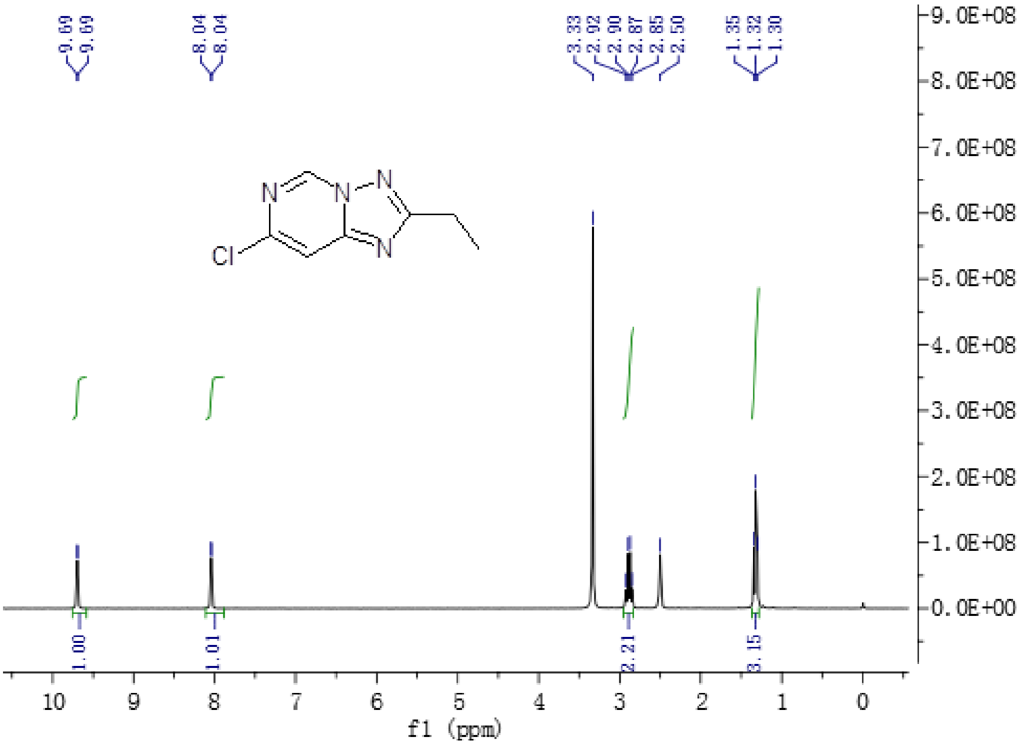

7-Chloro-2-ethyl-[1,2,4]triazolo[1,5-*c*]pyrimidine (6e)

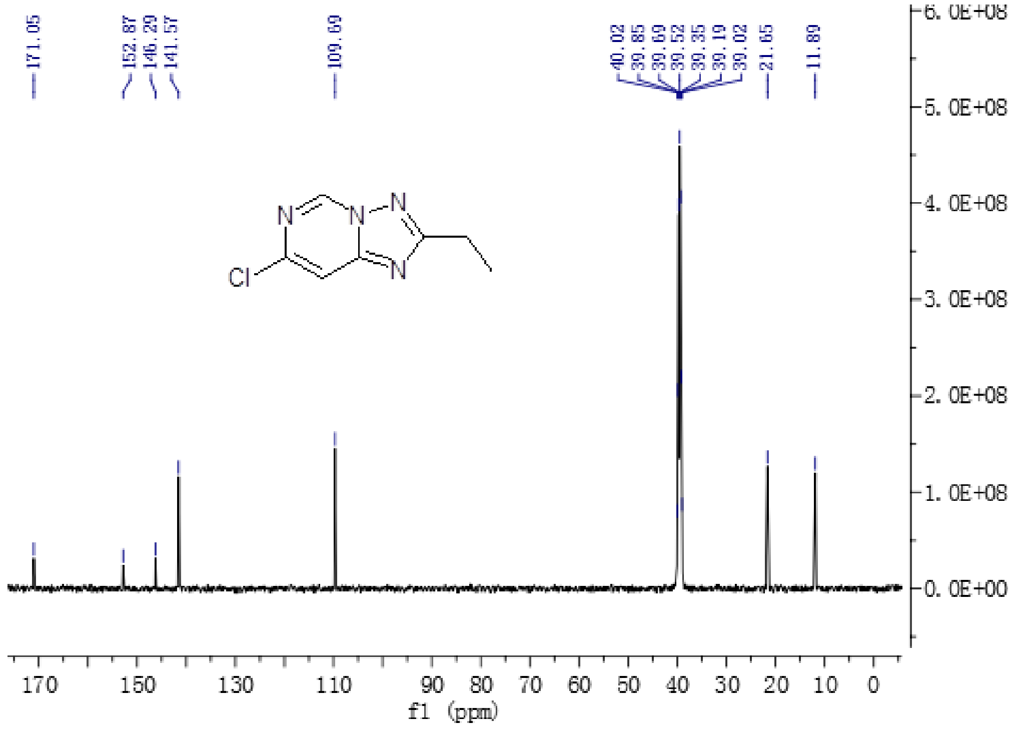

7-Chloro-5-methyl-2-phenyl-[1,2,4]triazolo[1,5-*c*]pyrimidine (6f)

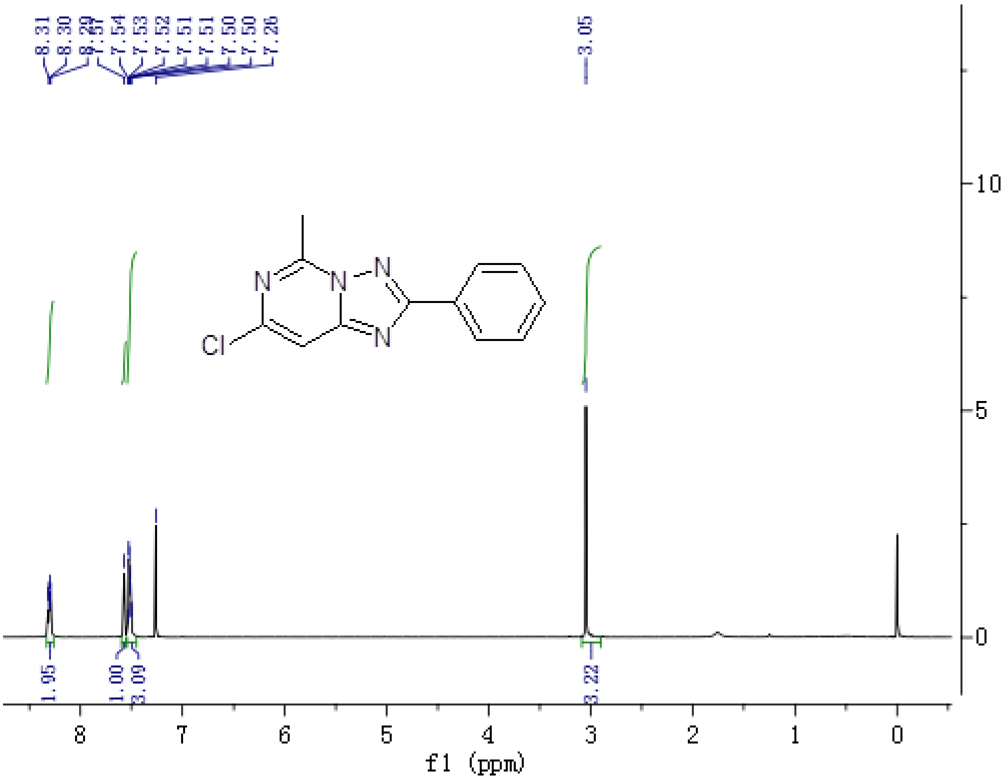

7-Chloro-5-methyl-2-phenyl-[1,2,4]triazolo[1,5-*c*]pyrimidine (6f)

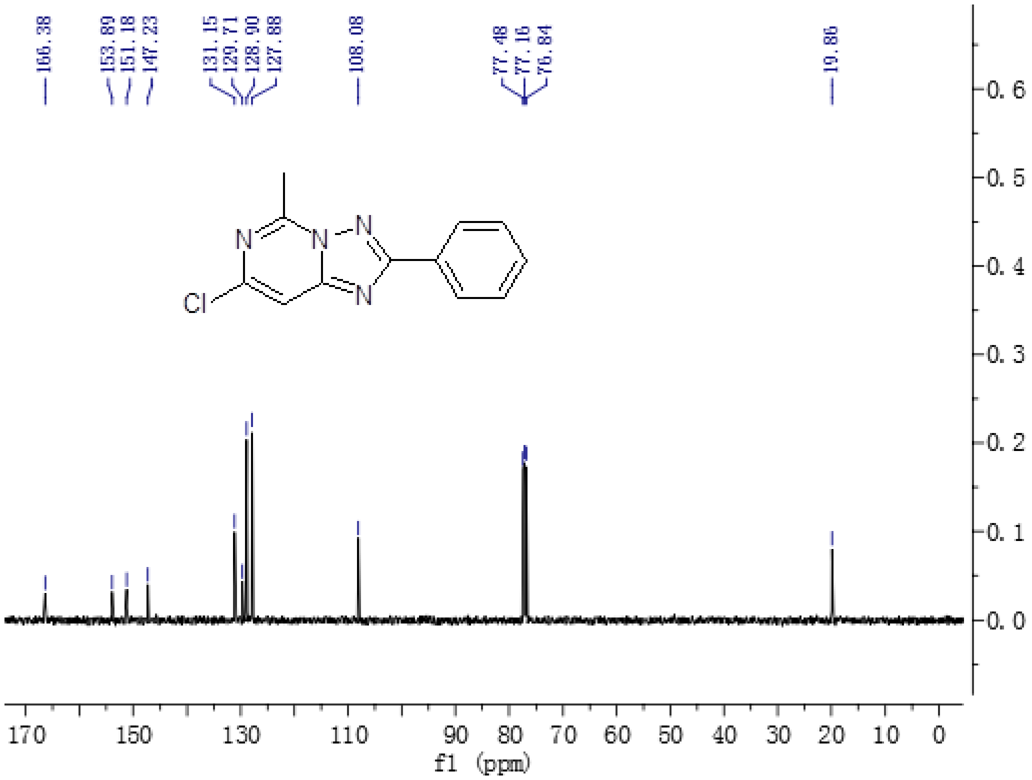

7-Chloro-2-(2-chlorophenyl)-5-methyl-[1,2,4]triazolo[1,5-*c*]pyrimidine (6g)

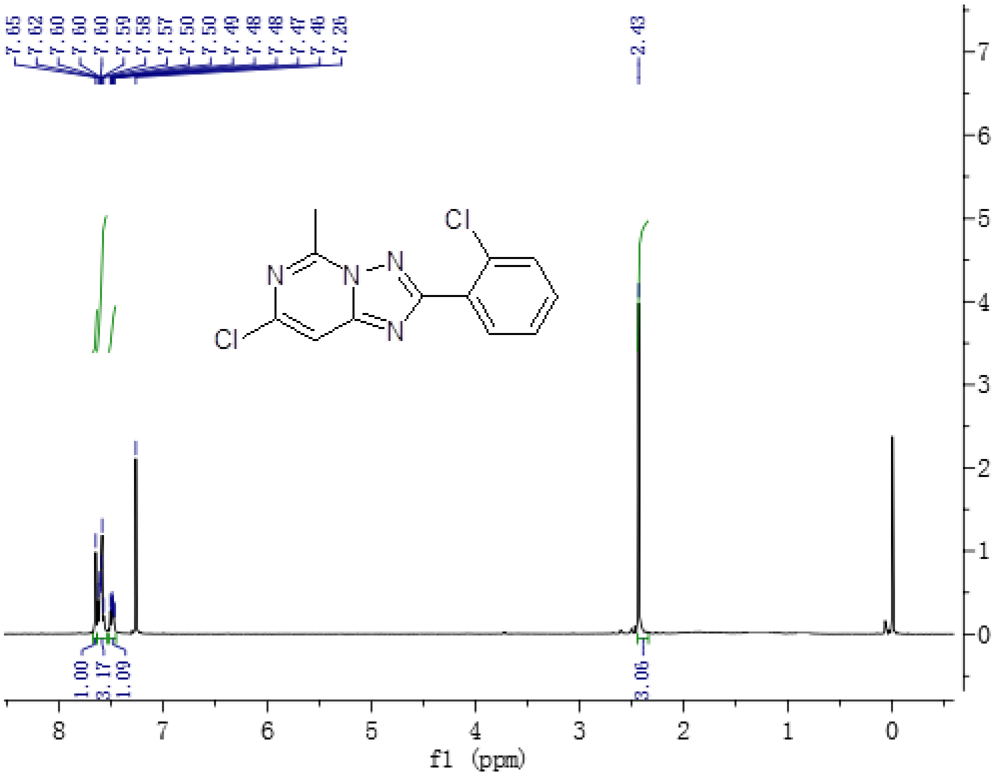

7-Chloro-2-(2-chlorophenyl)-5-methyl-[1,2,4]triazolo[1,5-*c*]pyrimidine (6g)

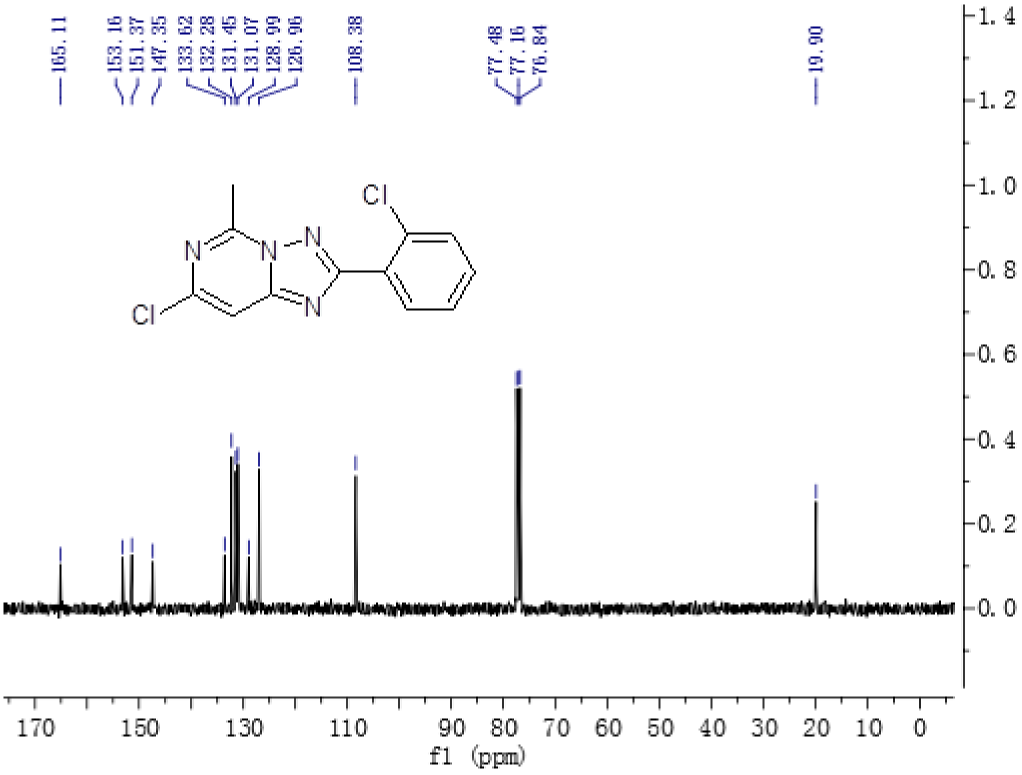

7-Chloro-2-(2-furanyl)-5-methyl-[1,2,4]triazolo[1,5-*c*]pyrimidine (6h)

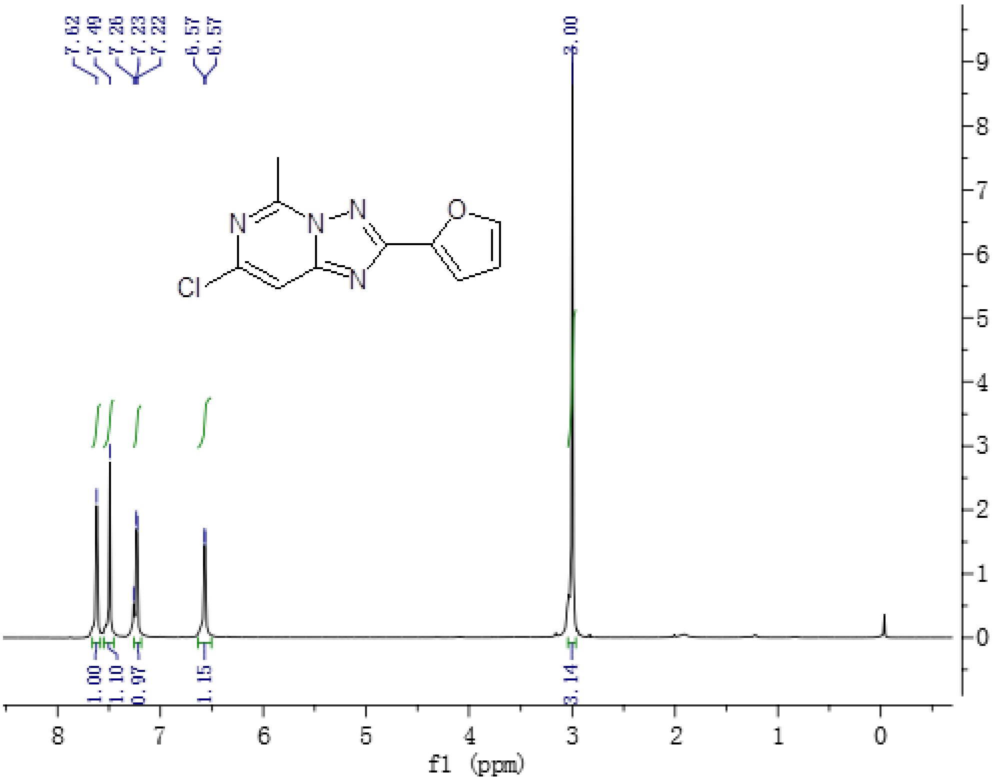

7-Chloro-2-(2-furanyl)-5-methyl-[1,2,4]triazolo[1,5-*c*]pyrimidine (6h)

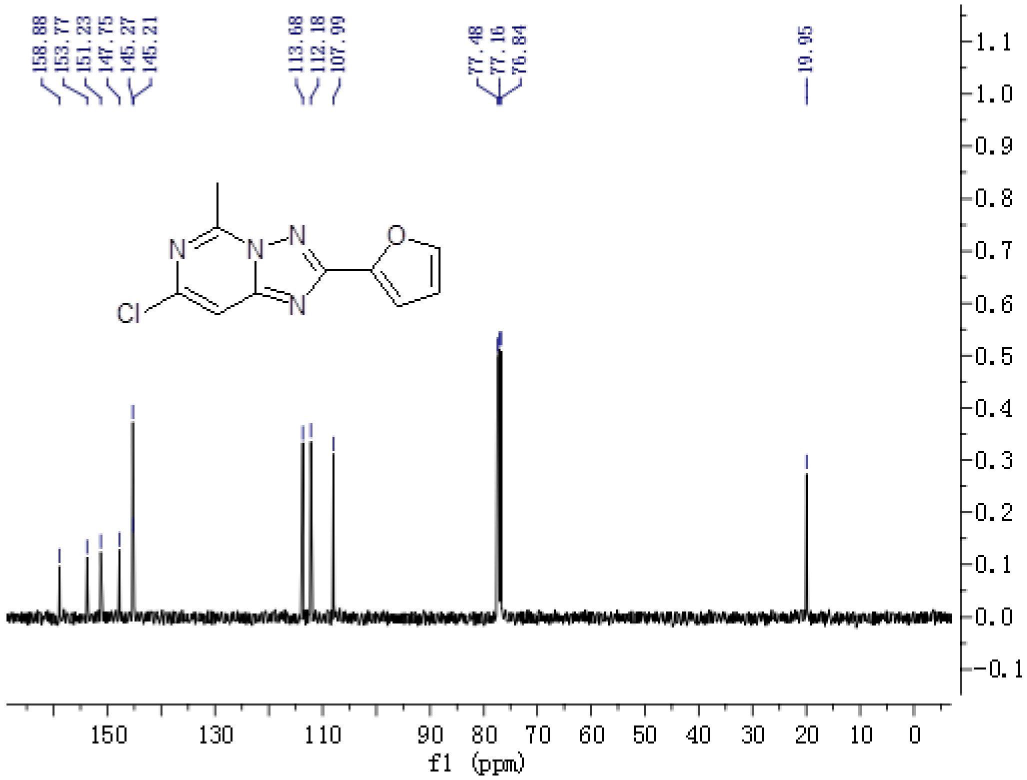

7-Chloro-2-(4-methoxyphenyl)-5-methyl-[1,2,4]triazolo[1,5-*c*]pyrimidine (6i)

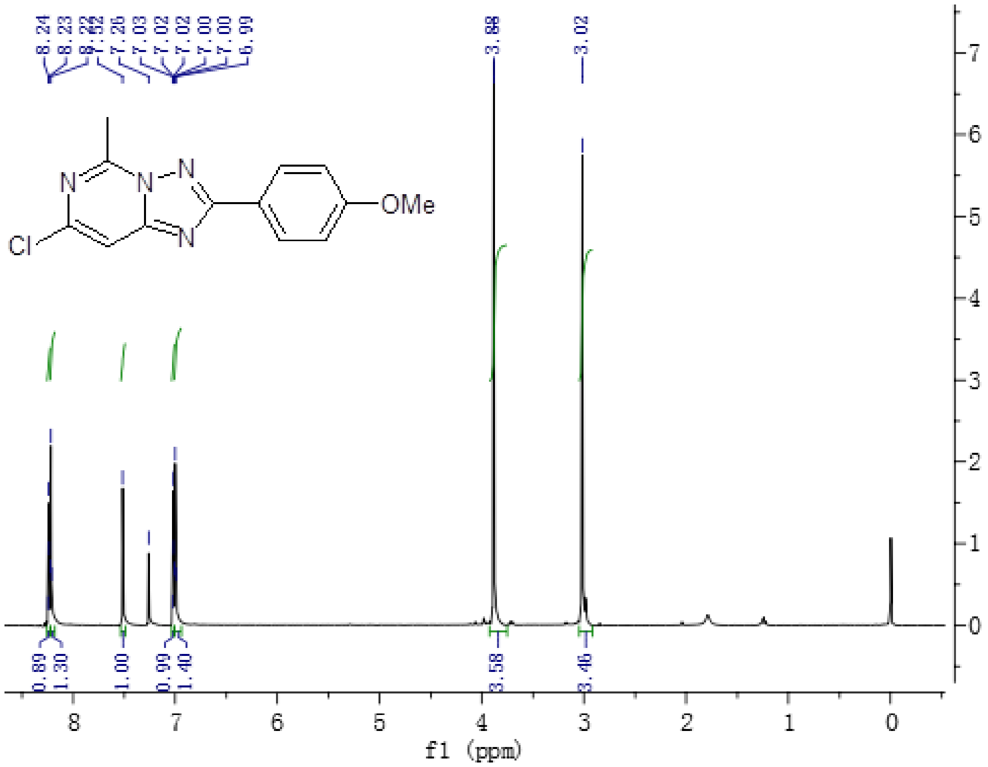

7-Chloro-2-(4-methoxyphenyl)-5-methyl-[1,2,4]triazolo[1,5-*c*]pyrimidine (6i)

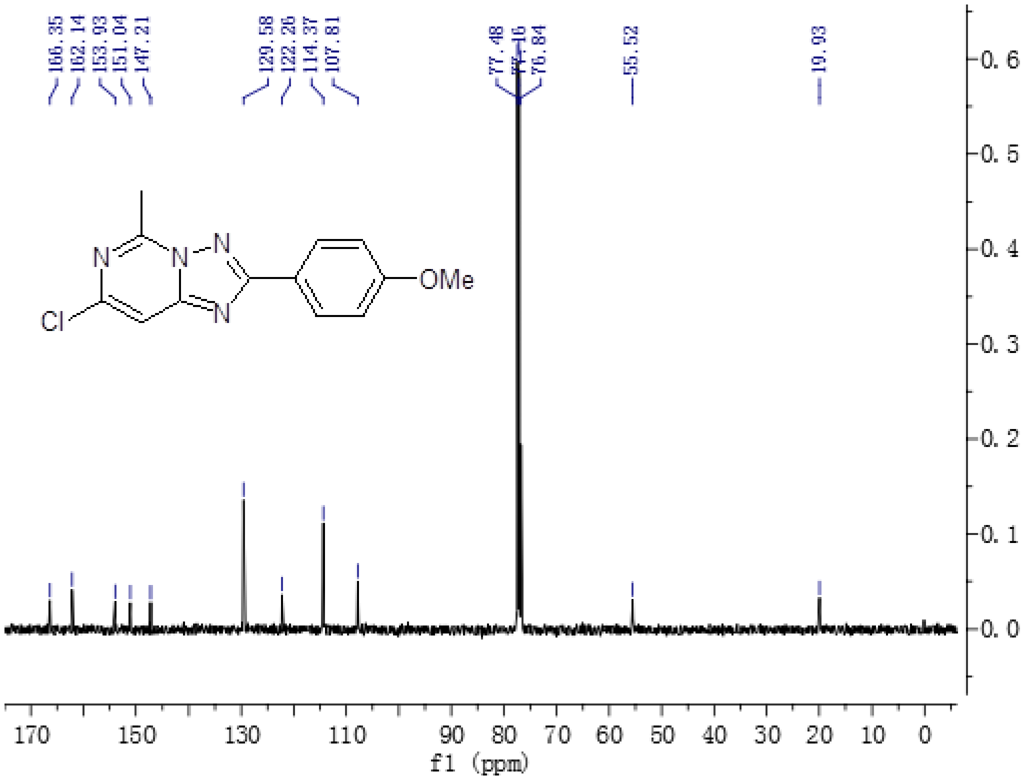

7-Chloro-2-ethyl-5-methyl-[1,2,4]triazolo[1,5-*c*]pyrimidine (6j)

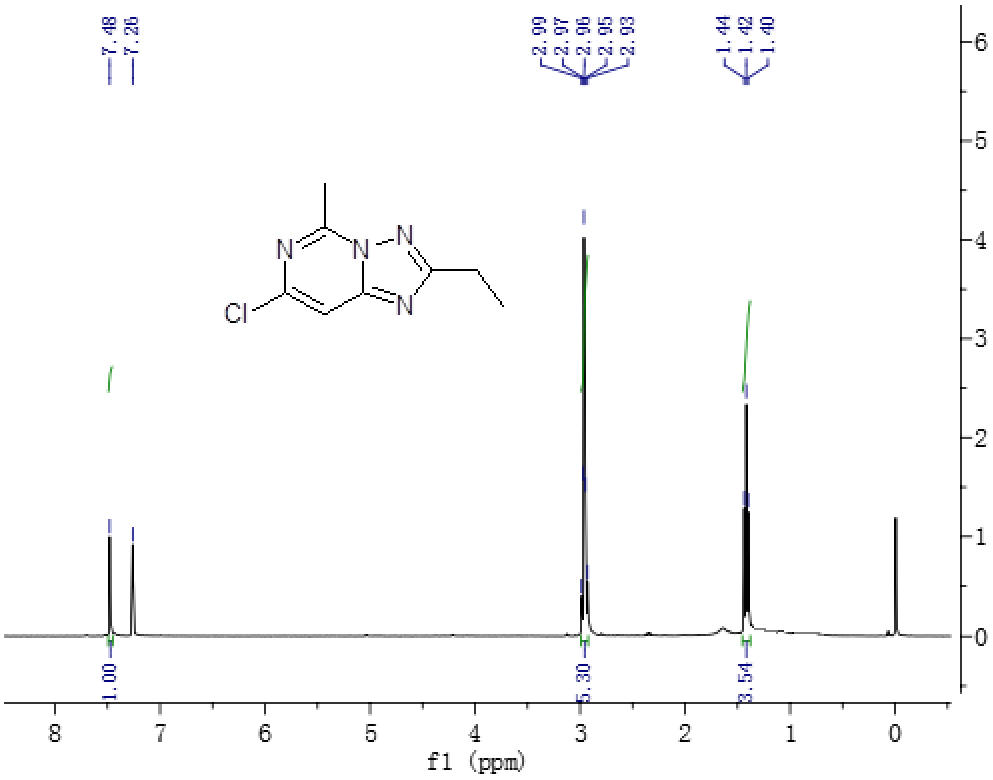

7-Chloro-2-ethyl-5-methyl-[1,2,4]triazolo[1,5-*c*]pyrimidine (6j)

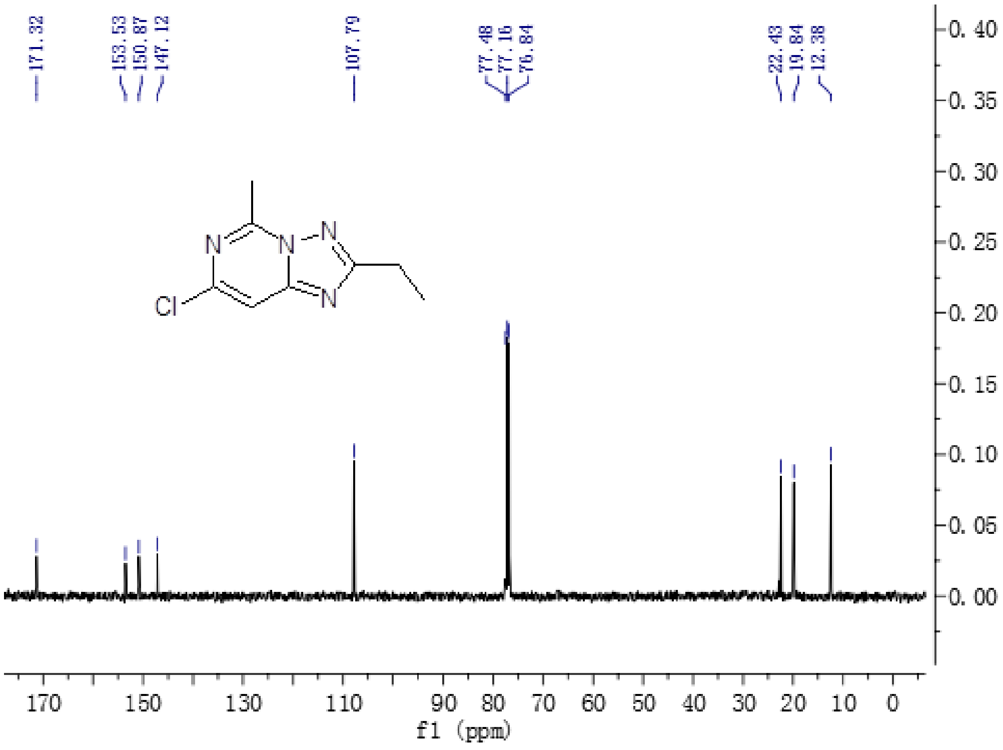

7-Chloro-2,5-diphenyl-[1,2,4]triazolo[1,5-*c*]pyrimidine (6k)

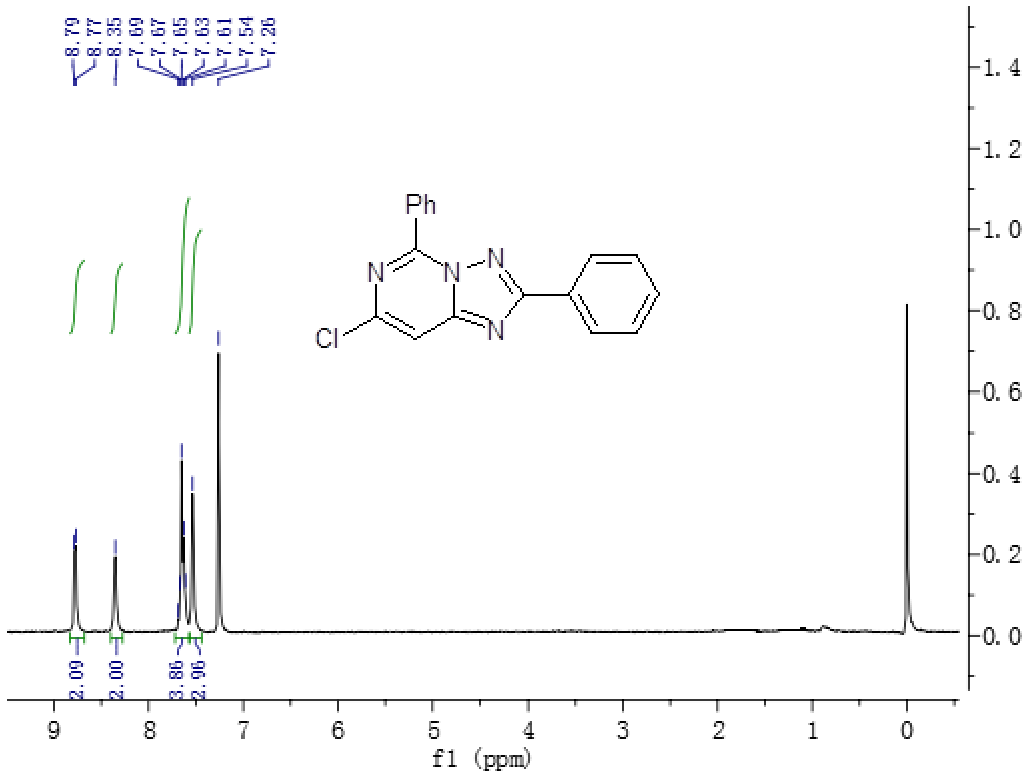

7-Chloro-2,5-diphenyl-[1,2,4]triazolo[1,5-*c*]pyrimidine (6k)

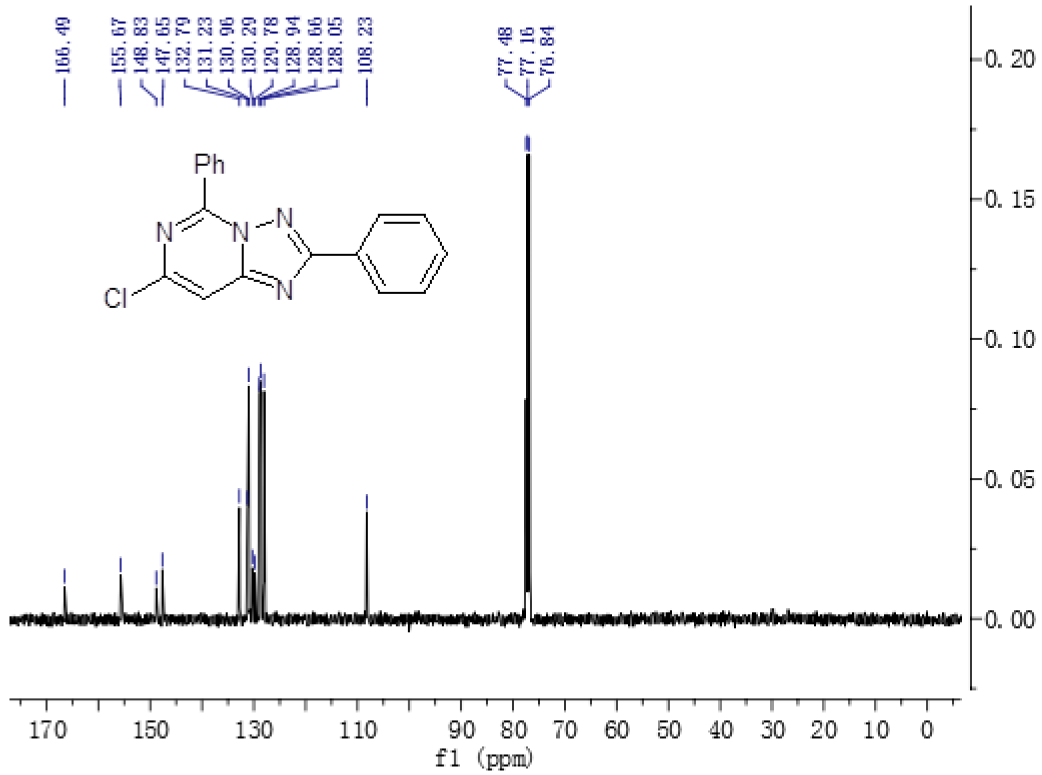

7-Chloro-2-(2-chlorophenyl)-5-phenyl-[1,2,4]triazolo[1,5-*c*]pyrimidine (6l)

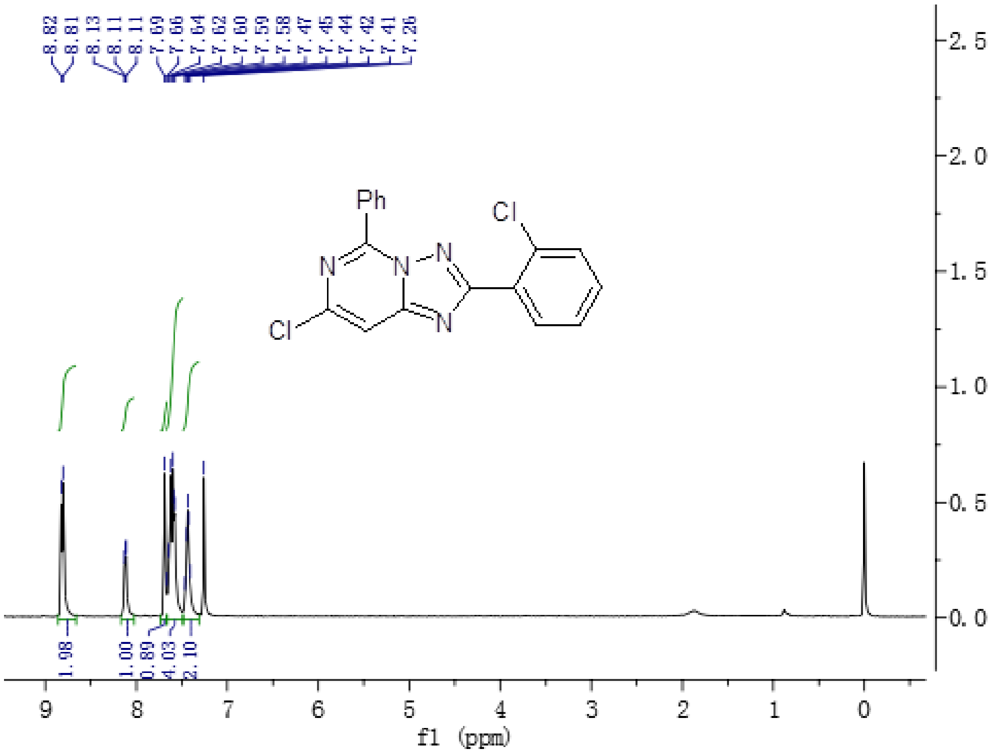

7-Chloro-2-(2-chlorophenyl)-5-phenyl-[1,2,4]triazolo[1,5-*c*]pyrimidine (6l)

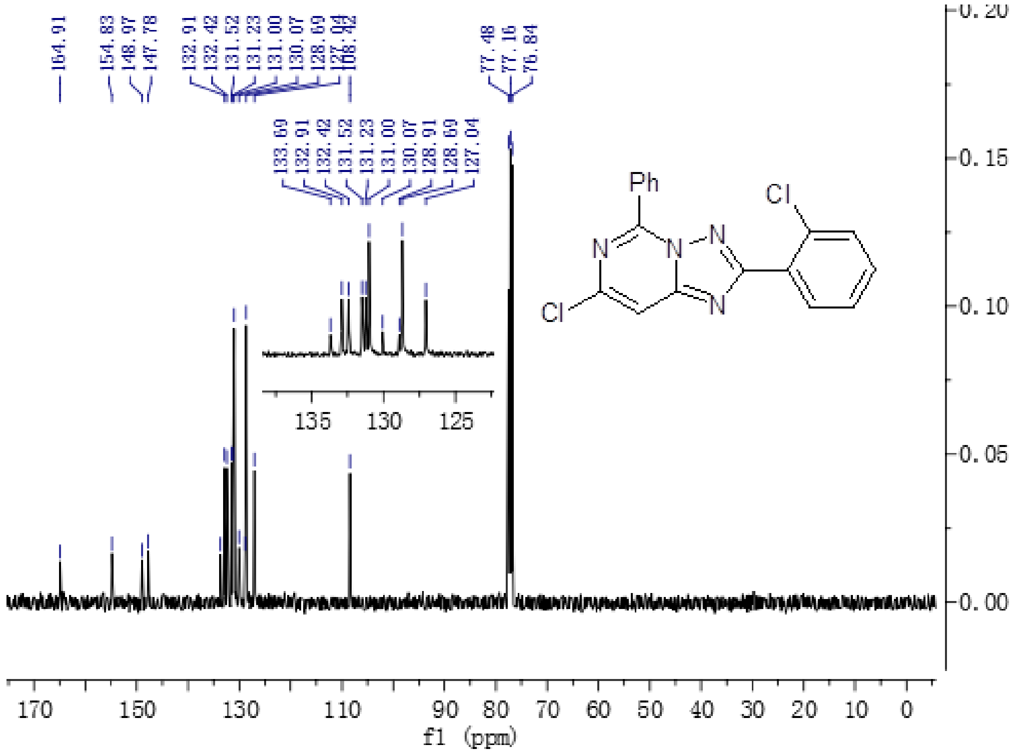

7-Chloro-2-(2-furanyl)-5-phenyl-[1,2,4]triazolo[1,5-*c*]pyrimidine (6m)

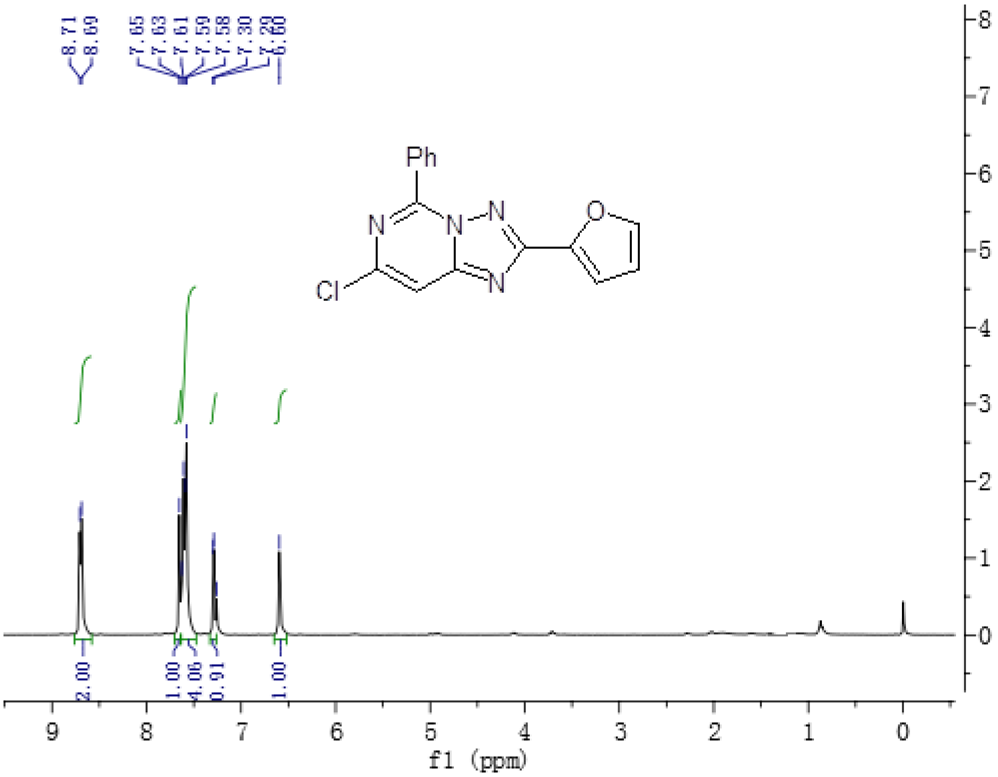

7-Chloro-2-(2-furanyl)-5-phenyl-[1,2,4]triazolo[1,5-*c*]pyrimidine (6m)

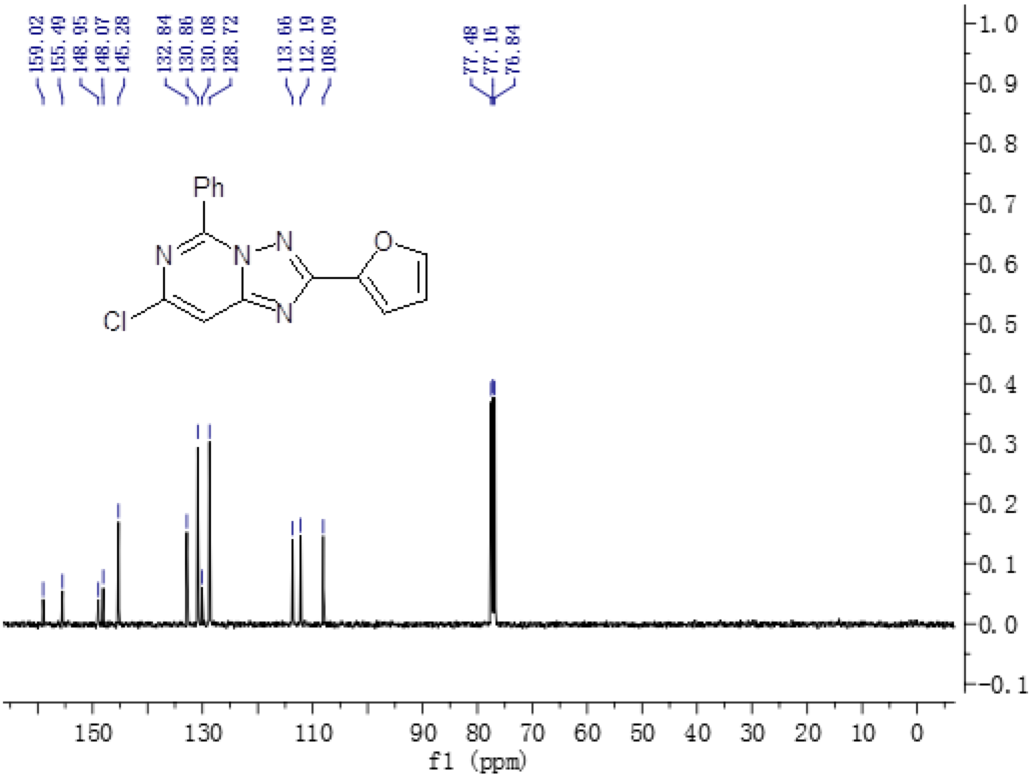

7-Chloro-2-(4-methoxyphenyl)-5-phenyl-[1,2,4]triazolo[1,5-*c*]pyrimidine (6n)

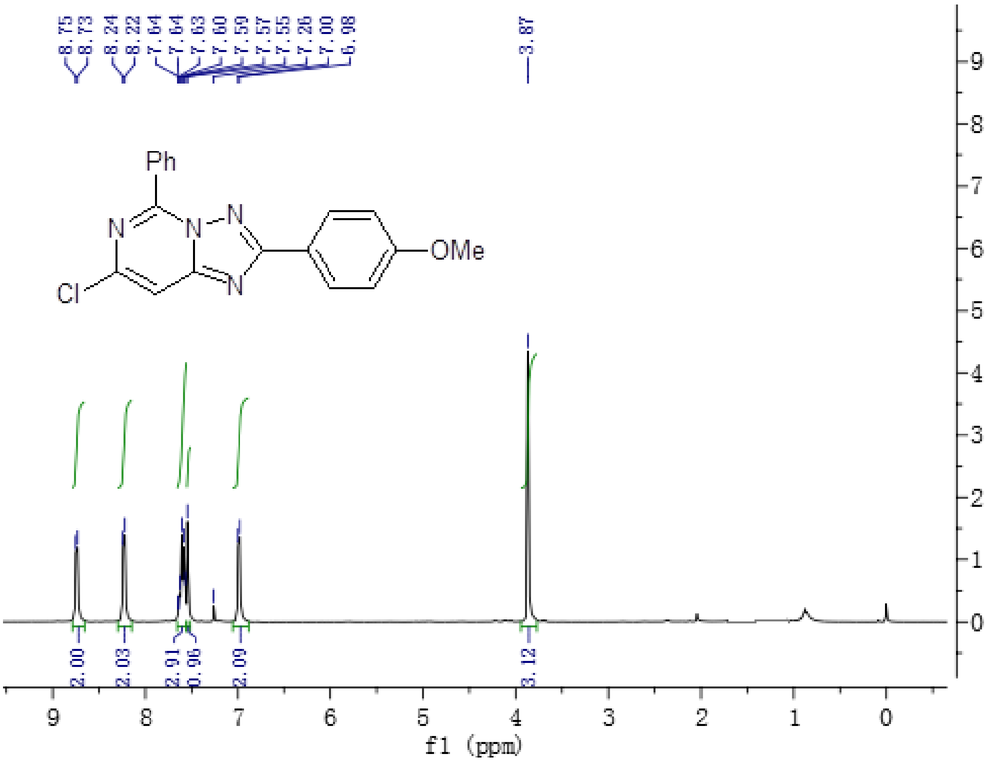

7-Chloro-2-(4-methoxyphenyl)-5-phenyl-[1,2,4]triazolo[1,5-*c*]pyrimidine (6n)

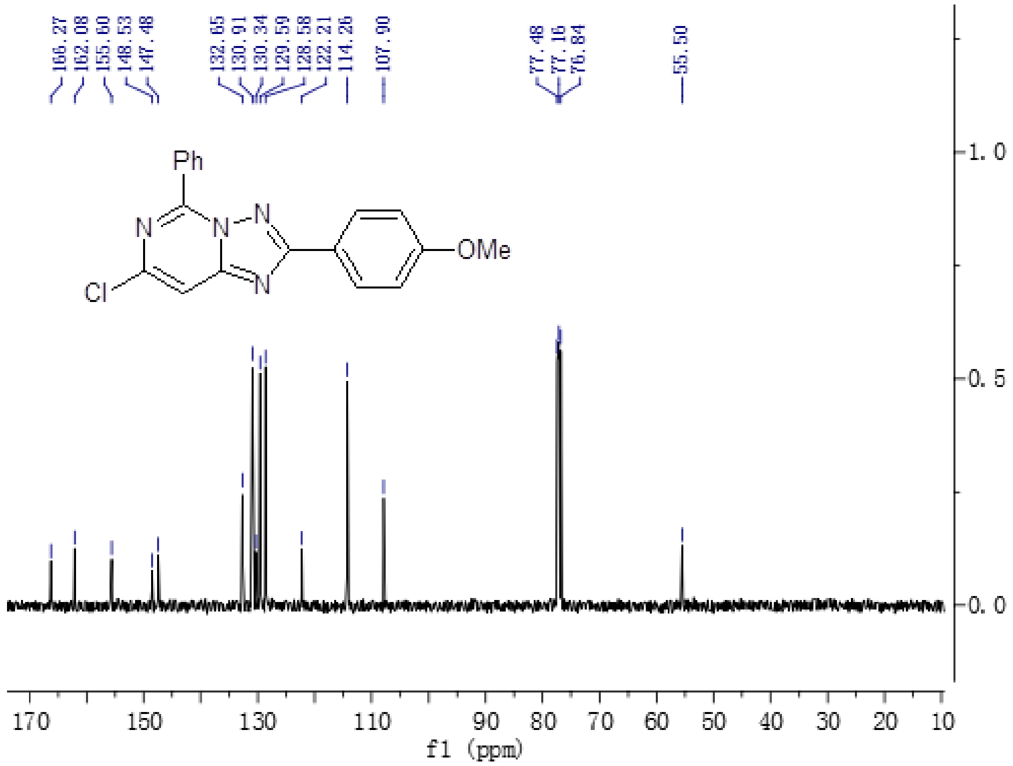

7-Chloro-2-ethyl-5-phenyl-[1,2,4]triazolo[1,5-*c*]pyrimidine (6o)

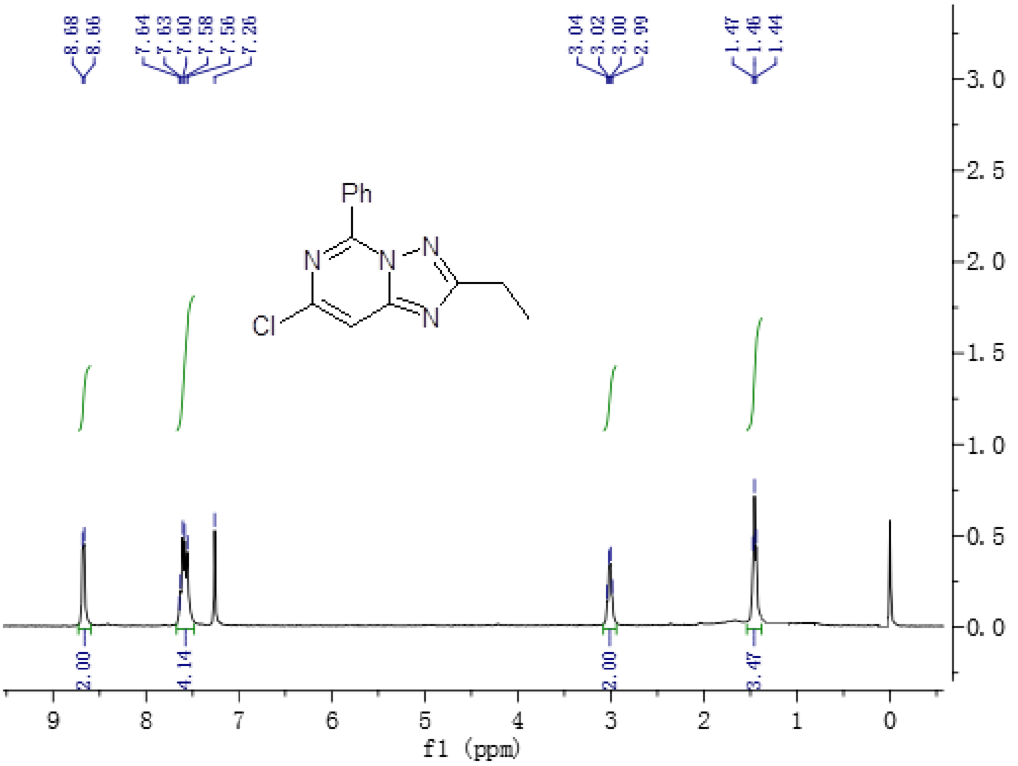

7-Chloro-2-ethyl-5-phenyl-[1,2,4]triazolo[1,5-*c*]pyrimidine (6o)

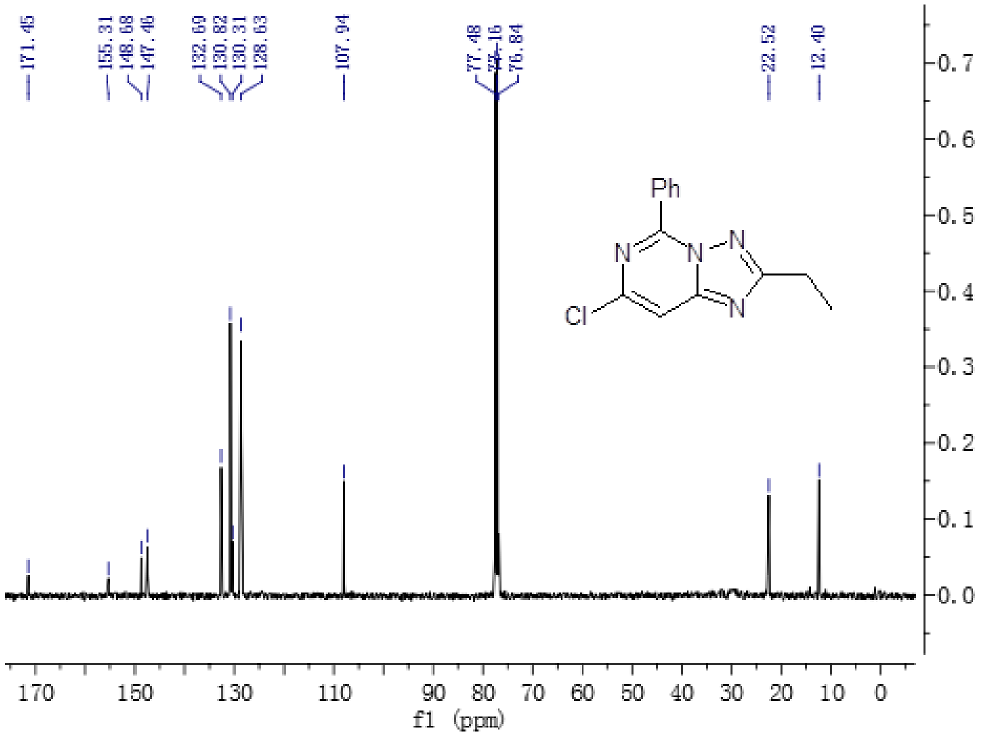

Supplement: File 2 — NMR spectral data for unknown compounds. [file Beilstein_J_Org_Chem-09-2629-s002.pdf]
